# Contrasting methane emissions from upstream and downstream rivers and their associated subtropical reservoir in eastern China

**DOI:** 10.1038/s41598-019-44470-2

**Published:** 2019-05-30

**Authors:** Le Yang

**Affiliations:** Zhejiang Academy of Forestry, Hangzhou, 310023 China

**Keywords:** Climate sciences, Biogeochemistry, Risk factors

## Abstract

Subtropical reservoirs are an important source of atmospheric methane (CH_4_). This study investigated the spatiotemporal variability of bubble and diffusive CH_4_ emissions from a subtropical reservoir, including its upstream and downstream rivers, in eastern China. There was no obvious seasonal variation in CH_4_ emissions from the main reservoir, which increased slightly from the first half year to the next half year. In the upstream river, CH_4_ emissions were low from February to June and fluctuated widely from July to January due to bubble activity. In the downstream river, CH_4_ emissions were lowest in February, which was possibly influenced by the low streamflow rate from the reservoir (275 m^3^ s^−1^) and a short period of mixing. There was spatial variability in CH_4_ emissions, where fluxes were highest from the upstream river (3.65 ± 3.24 mg CH_4_ m^−2^ h^−1^) and lowest from the main reservoir (0.082 ± 0.061 mg CH_4_ m^−2^ h^−1^), and emissions from the downstream river were 0.49 ± 0.20 mg CH_4_ m^−2^ h^−1^. Inflow rivers are hot spots in bubble CH_4_ emissions that should be examined using field-sampling strategies. This study will improve the accuracy of current and future estimations of CH_4_ emissions from hydroelectric systems and will help guide mitigation strategies for greenhouse gas emissions.

## Introduction

Hydropower has historically been regarded as a clean energy source, however, the view is challenged by a growing body of research that considers hydroelectric reservoirs to be carbon sources. For example, Deemer *et al*. (2016) showed that CH_4_ emissions were responsible for the majority of the radiative forcing from reservoir water surfaces, totalling approximately 80% over a 100-year timescale^1^. Greenhouse gas emission data are available for 36 Asian reservoirs, of which CH_4_ emission flux data have been reported for three reservoirs in China, including Three Gorges^2,3^, Ertan^4^, and Miyun^5^. However, there are more than 98,000 dams of varying sizes and 142 large-size hydroelectric reservoirs in a range of geographical regions and climate zones in China, excluding dams that are either under construction or planned, from which CH_4_ emission fluxes remain to be assessed.

Diffusive flux and gas bubble flux are the primary pathways for CH_4_ emissions from open water areas of reservoirs^6^. Ebullition has been shown to be the dominant CH_4_ emission pathway, albeit episodic^7^, but pulses of gas bubbles often occur during periods of rapidly falling barometric pressure in lakes, reservoirs, and peatland^7–10^. Ebullitive CH_4_ flux is reported to be 1–3 orders of magnitude greater than diffusive CH_4_ flux^11,12^, and high ebullitive CH_4_ flux, observed in shallow water, river deltas, and inflow rivers^11–13^, is shown to be influenced by allochthonous organic carbon input and burial^14^. Chamber methods were used to measure CH_4_ emission flux from three large reservoirs in China, where the total CH_4_ emission flux (diffusion + ebullition) was measured across the water-air interface^2–5^; however, it is likely that these studies did not capture the magnitude of bubble CH_4_ flux.

Subtropical reservoirs are strong atmospheric CH_4_ sources with strong spatial variability^15^. Such variations are presumably caused by changes in hydrological characteristics from impoundment. For example, increases in the water level, reduced water velocity, and flooded soils near the bank impact CH_4_ emissions from a new reservoir compared with the original river^7,16^. Similarly, CH_4_ emission levels from outlets downstream and inflow rivers upstream of reservoirs were distinct from those of the reservoir water^7,17,18^, due to variability in hydrological variables, such as water velocity and depth^2^, and dam operation strategy^19^. Temporal variability in CH_4_ emissions has been attributed to changes in temperature, water column mixing, dissolved oxygen (DO) concentration, and other environmental variables, including retention time and benthic metabolism^2,20,21^. For example, CH_4_ emissions were greater in summer than in other seasons at the Three Gorges Reservoir and were regulated by temperature, DO, and water velocity^2^, whereas they were only regulated by temperature at three lakes (Följesjön, Erssjön, and Skottenesjön) in southwest Sweden^20^. Analysis of these differences in effects of environmental factors on spatiotemporal variability in CH_4_ emissions from reservoirs may result in more accurate estimates of the total CH_4_ emissions than previously determined.

Emissions occur from rivers downstream of reservoirs, due to degassing fluxes at turbines and spillways. A large quantity of CH_4_ emits in the downstream river when the hypolimnion water passes through turbines and spillways because of the differences in temperature and pressure. The rapid stream of water increases the water current velocity, which enhances the gas transfer velocity at the air-water interface and improves downstream CH_4_ emission flux^22^. 50% of the total CH_4_ emissions recorded downstream from the Balbina Reservoir in Brazil^18^ represented approximately 30% of the total greenhouse gas emissions from the eight reservoirs in the dry tropical biome region of the country^23^, whereas downstream emissions accounted for 10% of the total CH_4_ emissions from the Nam Theun 2 Reservoir in Laos^24^.

In this study, we compared CH_4_ emissions from a reservoir with sites upstream and downstream to quantify spatial variations in CH_4_ emissions to ensure a more accurate estimation of CH_4_ emissions from hydroelectric reservoir systems. Specifically, we tested the hypothesis that upstream and downstream CH_4_ emissions are greater than from a reservoir.

## Results

### Temporal variation in ebullitive CH_4_ emissions

**Figure 1:**
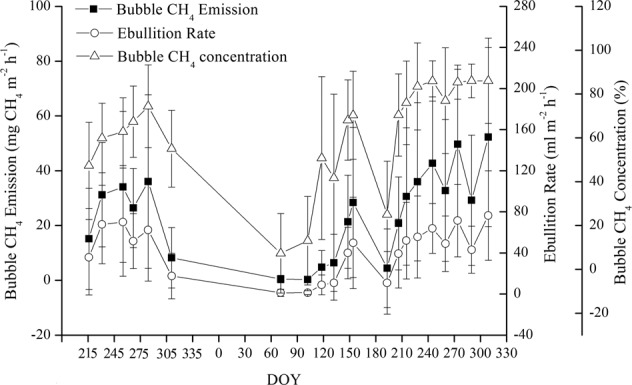
Mean ebullition rates, bubble CH_4_ emissions flux, and CH_4_ concentrations recorded from the inflow river. DOY: day of year, from 3 August 2016. Bars are ± SE, n = 3.

There were similar seasonal patterns of ebullition rate, bubble CH_4_ emission flux, and bubble CH_4_ concentration, all of which were lower in spring than in summer and autumn (Fig. 1). Mean ebullition rate from the upstream river was 39.93 ± 24.3 ml m^−2^ h^−1^ (range: 1.17–76.4 ml m^−2^ h^−1^), mean bubble CH_4_ flux rate was 22.62 ± 15.1 mg CH_4_ m^−2^ h^−1^ (range: 0.31–52.27 mg CH_4_ m^−2^ h^−1^), and mean CH_4_ concentration by volume in the collected gas was 59.04 ± 23.3% (range: 7.32–86.03%). Ebullitive CH_4_ flux positively correlated with the ebullition rate (*R*^2^ = 0.92, *P* < 0.001) and bubble CH_4_ concentration (*R*^2^ = 0.76, *P* < 0.001, see Supplementary Fig. S3).

### Temporal variation in diffusive CH_4_ emissions

**Figure 2:**
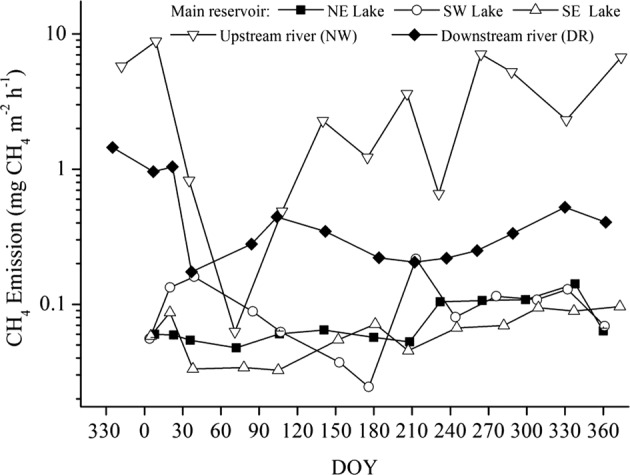
Seasonal dynamics in the average diffusive CH_4_ emissions, measured using floating chambers, from the different regions of Xin’anjiang Reservoir.

CH_4_ emissions from the upstream river (NW) were low from February to June, but increased and fluctuated from July to January (Fig. 2). Furthermore, on a monthly scale, mean diffusive CH_4_ fluxes during the sampling period were similar and generally constant over time among the three areas of the main reservoir; however, fluxes peaked in the southwest (SW) lake on 1 August (DOY: 213) and 8 February (DOY: 39; Fig. 2). On a seasonal scale, there were similar seasonal patterns in CH_4_ fluxes among the three areas of the reservoir, where they were lowest in the spring and highest in the autumn (see Supplementary Fig. S4). Mean CH_4_ fluxes on the northeast (NE), SW, and southeast (SE) lakes in the next half year were 1.72, 1.54, 1.57 times as many as those in the first half of year, respectively. In addition, there was some temporal variation in mean CH_4_ emissions downstream of the reservoir (DR), where it was highest in December 2014 and lowest in February 2015; otherwise, emissions were generally constant (Fig. 2).

### Spatial variation in CH_4_ emissions

**Figure 3:**
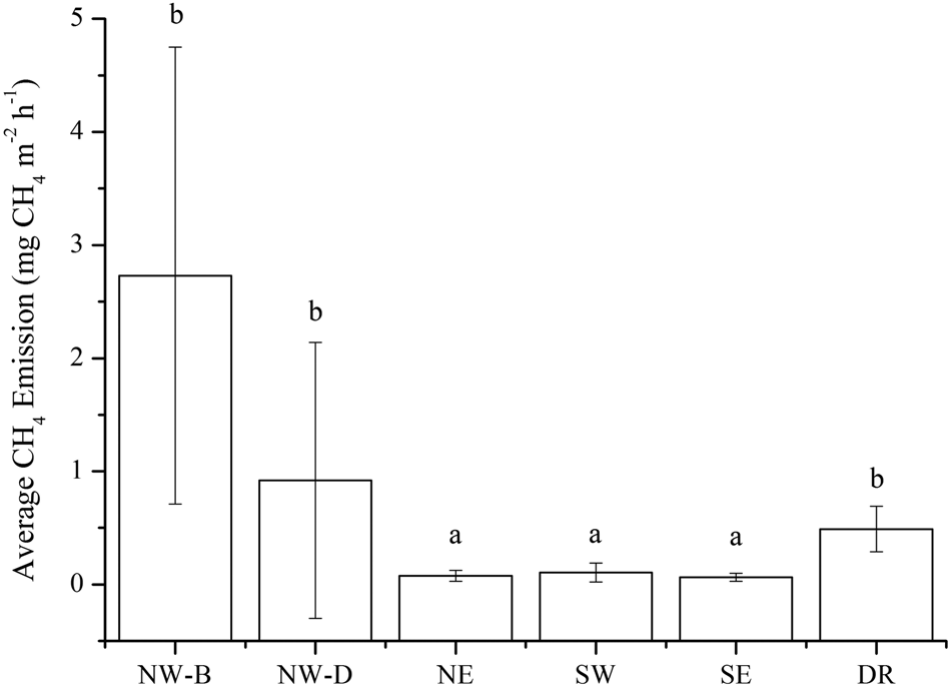
Mean CH_4_ emissions from the reservoir and the upstream and downstream rivers. NW-B, bubble emissions from the northwest transect (upstream); NW-D: diffusive emissions from the northwest transect. NE, northeast lake; SW, southwest lake; SE, southeast lake; DR, downstream river. Different small letters indicate the differences in mean CH_4_ emissions flux among the sampling areas at *P* = 0.05.

Mean flux in CH_4_ emissions from the upstream river was 3.65 ± 3.24 mg CH_4_ m^−2^ h^−1^, whereas mean bubble CH_4_ flux (NW-B) was 2.73 ± 2.02 mg CH_4_ m^−2^ h^−1^ and diffusive CH_4_ flux (NW-D) was 0.92 ± 1.22 mg CH_4_ m^−2^ h^−1^ (Fig. 3). Although there were no bubble CH_4_ emissions in the reservoir or the downstream river, the mean diffusive CH_4_ emission flux in the reservoir was 0.082 ± 0.061 mg CH_4_ m^−2^ h^−1^ (NE: 0.076 ± 0.049 mg CH_4_ m^−2^ h^−1^, SW: 0.106 ± 0.083 mg CH_4_ m^−2^ h^−1^, and SE: 0.064 ± 0.034 mg CH_4_ m^−2^ h^−1^), which was lower than in the downstream river, where it was 0.49 ± 0.20 mg m^−2^ h^−1^ (Fig. 3). Mean diffusive CH_4_ emissions from the upstream and downstream rivers were higher than those from the reservoir by a factor of 11 and 6, respectively (Fig. 3).

There was no significant difference in mean CH_4_ emissions from the marginal to pelagic zones among the three sampling areas of the reservoir (see Supplementary Fig. S5C–E); however, the mean CH_4_ emissions from the nearest sampling-point in the downstream river (DRP1: 0.78 ± 0.44 mg CH_4_ m^−2^ h^−1^) were significantly higher than those from the second nearest sampling-point (DRP2: 0.34 ± 0.30 mg CH_4_ m^−2^ h^−1^; *P* < 0.001; see Supplementary Fig. S5A), and the average CH_4_ emissions from the pelagic zones of the upstream river were significantly higher than those from the marginal zone (see Supplementary Fig. S5B).

### Effects of temperature and wind speed on CH_4_ emissions

CH_4_ flux from the reservoir was positively correlated with wind speed and air-water temperature difference, whereas CH_4_ flux from the downstream river was positively correlated with air-water temperature difference (see Supplementary Tables S1 and S2).

## Discussion

### Comparison of CH_4_ emissions with other reservoirs

Average CH_4_ emissions from the main reservoir (0.082 ± 0.061 mg CH_4_ m^−2^ h^−1^) are lower than those from the other temperate and subtropical reservoirs listed in Table 1, except for Douglas Lake, which is presumably due to the deep, oxic conditions and clean water quality in Xin’anjiang Reservoir^25,26^. The mean CH_4_ emissions from the upstream river in the study are comparable to that from Three Gorges Reservoir (2.72 mg CH_4_ m^−2^ h^−1^), which is one order of magnitude greater than that from Eguzon Reservoir (0.24 mg CH_4_ m^−2^ h^−1^), but significantly lower than those from Australian reservoirs and an agriculturally impacted reservoir in the United States, due to the differences in bubble activity (Table 1). The heterogeneity, specifically, differences in ebullition frequency and ebullition magnitudes, contribute to the variability in average CH_4_ fluxes observed among the reservoirs^12^. Frequency of bubble occurrence upstream of the reservoir is low (16.2%), and the average ebullitive CH_4_ emission level is 16.83 mg CH_4_ m^−2^ h^−1^ (Table S4), which is one order of magnitude lower than the ebullition magnitudes in William H. Harsha Lake (130.7 mg CH_4_ m^−2^ h^−1^), Gold Creek (172.4 mg CH_4_ m^−2^ h^−1^), and Little Nerang Dam (165.7 mg CH_4_ m^−2^ h^−1^; Table 1). A large number of bubbles contribute to the extremely high CH_4_ emissions from the inflow rivers in the three reservoirs^15,27–29^. In regard to the downstream river of the reservoir, it has comparable CH_4_ emissions levels with the other listed reservoirs in Table 1.

**Table 1:** Literature review of CH_4_ emissions from temperate and subtropical reservoirs.

Country	Reservoir	CH_4_ Flux (mg CH_4_ m^−2^ h^−1^)			Refs
		Upstream river*	Open water area	Downstream river	
China	Xin’anjiang	2.73 ± 2.02 (B) 0.92 ± 1.22 (D)	0.082 ± 0.061	0.49 ± 0.20	This study
	Three Gorges	2.72 ± 1.98	0.23 ± 0.40	0.26 ± 0.16	^2, 3^
	Ertan		0.12 ± 0.063		^4^
	Miyun		0.30 ± 0.31		^5^
	16 reservoirs in Chongqing		0.63 ± 0.89		^50^
USA	William H. Harsha Lake	130.72 ± 27.50	9.77 ± 2.00		^27^
	Douglas Lake	0.018 (D)	0.017 ± 0.012		^51^
	Eagle Creek		0.44 ± 0.73		^52^
	6 reservoirs in Western US		0.13–0.40		^53^
Australia	Gold Creek	172.36 ± 24.72	12.35 ± 6.36		^28^
	Little Nerang Dam	165.70 ± 236.43	7.70 ± 19.38		^29^
Laos	Nam Leuk		1.68 ± 2.68		^54^
	Nam Ngum		0.13 ± 0.13		^54^
	Nam Theun 2		1.0–2.67	Below the powerhouse: 8.0 ± 14.7 Below the Nakai Dam: 0.93–2.2	^24, 33^
France	Eguzon	0.24 ± 0.56 (B) 2.2 ± 3.2 (D)	0.4 (0–2.67)	0.68 ± 0.68	^55^

*CH_4_ flux in upstream river: B: Bubble emission, D: Diffusive emission.

### Seasonal variation in CH_4_ emissions

**Figure 4:**
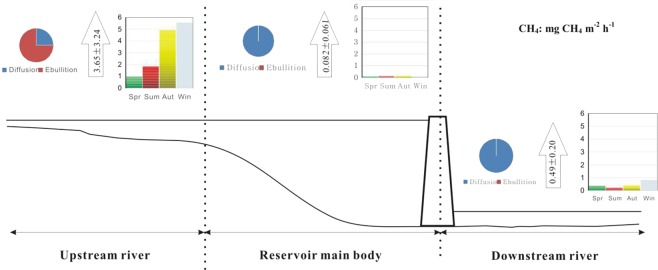
Schematic diagram of the spatiotemporal variability in CH_4_ emissions from Xin’anjiang Reservoir.

In the upstream river, CH_4_ emissions in autumn and winter are higher than those in spring and summer (Fig. 4), due to the differences in the frequency of bubbles (22.6% versus 8%), but the differences do not reach a significant level (p > 0.05) after performing a one-way ANOVA test. However, the results measured by the bubble traps indicate that the bubble CH_4_ emissions in summer and autumn are significantly higher than those in spring (Fig. 1). One of the major differences between the two methods is the duration of the measurement. The measurements using bubble traps were performed over 20–33-h periods, whereas chamber measurements were conducted for 20–30 min only. The floating chambers captured both ebullition and diffusive gas emissions^27^, whereas only CH_4_ ebullition fluxes were collected using bubble traps^8^. However, the average ebullitive CH_4_ flux (22.62 ± 15.1 mg CH_4_ m^−2^ h^−1^) measured using bubble traps was approximately 5 times higher than that measured using floating chambers (3.65 ± 3.24 mg CH_4_ m^−2^ h^−1^). These differences can be explained by the sudden release of bubbles on these rare occasions, which reveals strong spatiotemporal heterogeneities of the ebullition process because ebullition is highly sporadic and occurs during a very short period of time^7^. The measurements using floating chambers are conducted over a short period of time and a small surface might lead to an underestimation of this emission pathway if hot spots and hot moments are missed during the deployment of the chambers. Such a phenomenon is strongly smoothed when using bubble traps over longer periods of time than the typical floating chamber deployment time (20–33 h versus 20–30 min)^30^.

Another explanation for the differences in CH_4_ emissions from the upstream river is that they were measured in different years (2014–2015 versus 2016–2017). Admittedly, interannual variability in upstream CH_4_ emissions presumably caused unnecessary errors. However, if the average CH_4_ flux is calculated only from these bubble-captured chambers in the NW transect in 2014 and 2015, it is 16.83 ± 12.48 mg CH_4_ m^−2^ h^−1^, which is approximately 25% less than that measured by the bubble traps (22.62 ± 15.1 mg CH_4_ m^−2^ h^−1^) in 2016 and 2017. Although both the interannual variability and different methods contributed to variances, differences remained in CH_4_ emissions when the diffusive and ebullitive CH_4_ fluxes were synchronously measured due to episodic bubbles. Nevertheless, the partitioning of bubble and diffusive CH_4_ emissions is an uncertainty in this study.

We observed that mean CH_4_ emissions from the reservoir in the second half of the year were higher than in the first half of the year (Fig. 2); this was due, in part, to seasonal hydrological dynamics. Hydrology mediates many biogeochemical processes, such as O_2_ concentration and thermal stratification, in aquatic systems. Zhang *et al*. (2015) found that oxycline and thermocline progressively sank in Xin’anjiang Reservoir in the second half of the year^25^. Vertical transport of CH_4_ in the water column is typically limited by slow rates of diffusion through the thermocline or oxycline^31^, and thermal and DO stratification typically become weaker in the second half of year, presumably resulting in increases in CH_4_ flux at the air-water interface.

Xin’anjiang Reservoir is a thermal stratification lake characterised by a short mixing period in February and March^25^. However, the vertical distributions of CH_4_ and O_2_ concentrations, and temperature were not measured in this study, which failed to illuminate the temporal variability in CH_4_ emissions. Lake overturn is a hot moment that exhibits disproportionately high CH_4_ emissions and CH_4_ oxidation^32^. Many studies indicate that CH_4_ storage sharply decreases during seasonal overturn periods^32–35^, emitting 12–46% of the total CH_4_ to the atmosphere, whereas the remainder (54–88%) is consumed by methane-oxidizing bacteria^32,34,35^. Although a minor proportion of the storage CH_4_ was emitted to the atmosphere, the contribution to the annual diffusive CH_4_ emissions was still great^32,34,35^, and even extremely diffusive CH_4_ fluxes occurred^33^. However, CH_4_ emissions from the main reservoir did not show a pulse in February and May (Fig. 2), presumably because the low measurement frequency in our study did not capture the CH_4_ emission peaks. Thermal stratification and its impact on CH_4_ emissions is important to understanding the mechanisms of the spatial and temporal variability of CH_4_ emissions from reservoirs, which should be examined in the near future.

We recorded a clear peak in CH_4_ emissions (0.25 ± 0.15 mg CH_4_ m^−2^ h^−1^) on 1 August (DOY: 213) in the SW lake (Fig. 2), which was presumably due to the fluxes from the two marginal sampling points (SWP1 and SWP2) of 0.47 ± 0.11 mg CH_4_ m^−2^ h^−1^ and 0.33 ± 0.061 mg CH_4_ m^−2^ h^−1^, respectively (see Supplementary Table S7). The high CH_4_ fluxes from the marginal zone may be attributed to the decomposition of vegetation in the littoral zone when the water level increased to its highest point (104.4 m) in July (see Supplementary Fig. S2). It is likely that the gentle slopes that had adequate levels of soil on the banks of the SW transect permitted the growth of vegetation in the littoral zone during the spring when water levels were low, whereas the banks of the NE and SE lakes were steep and rocky and presumably less well vegetated. Similar peaks in CH_4_ emissions have also been reported from littoral zones of the Miyun and Three Gorges Reservoirs^5,36^.

We recorded another peak, albeit low (0.16 ± 0.097 mg CH_4_ m^−2^ h^−1^) on 8 February (DOY: 39) in the SW lake (Fig. 2), which was caused by strong winds. Gas samples were only collected from three of the five sampling points due to unstable safety conditions on the surface of the reservoir. Mean CH_4_ fluxes were 0.23 and 0.20 mg CH_4_ m^−2^ h^−1^ at SWD2 and SWD4 (see Materials and Methods), respectively, when the wind speed reached 8–10 m s^−1^, whereas the lowest CH_4_ flux at SWD5 (0.049 mg CH_4_ m^−2^ h^−1^) occurred in the central area of the reservoir due to the low wind speed (2.63 m s^−1^). Many studies support the opinion that CH_4_ emissions from water surfaces can be enhanced by strong wind speeds^15,22,33,37^.

Downstream CH_4_ emissions (including degassing at the turbines) have been found to be proportional to streamflow^19^. It is impossible to calculate the degassing emissions from the turbines at Xing’anjiang Dam based on the differences in CH_4_ concentrations between the water intake and water outlet below the dam because access is forbidden 500 m upstream and downstream of the dam due to safety concerns. However, measurements of CH_4_ emissions at four distances downstream of the dam, taken 13 times in 2015 (see Supplemental Table S9), were found to be at their lowest (0.17 ± 0.11 mg CH_4_ m^−2^ h^−1^) in February, which is presumably a result of a low discharge flow rate (275 m^3^ s^−1^). Another possible explanation for the low flux is related to the lake overturn phenomenon in February^25^. Most of the CH_4_ stored in the hypolimnion is oxidized or released to the atmosphere during overturn periods^32–35^, and a very small fraction of the original quantity of CH_4_ remains in the water column^32^; thus, a low CH_4_ flux level was measured in the downstream river in February (Fig. 2).

### Spatial variation in CH_4_ emissions

Upstream CH_4_ emissions are hot spots because they exhibit disproportionately high ebullitive CH_4_ emissions relative to the surrounding matrix^38^. Upstream river CH_4_ emission dynamics are predominantly influenced by bubbles since the peaks in the CH_4_ emissions flux (Fig. 2) are driven by bubbles (see Supplementary Table S4). In contrast to other studies^21,24^, we found that bubbles occurred in the deep-water zone (>10 m) rather than in the shallow zone (<5 m), and we suggest that the high ebullitive CH_4_ emissions from deep water zones are related to heterogeneous sediment accumulation^12,13^ because little or no sediment accumulates along reservoir margins^39^.

The average CH_4_ emission rate at the upstream site (NW) was one to 2 orders of magnitude greater than the other sites (Figs 2 and 3), highlighting the importance of identifying ebullition hot spots to improve total emissions estimates^27^. The results supported our hypothesis that CH_4_ emissions are higher in rivers upstream and downstream of the reservoir than in the main reservoir (Fig. 3), where high CH_4_ emissions from the upstream river were mediated by bubbles (Figs 1 and 3, see Supplementary Table S4). The CH_4_ in the gas bubbles can escape oxidation during transport through the water column as CH_4_ moves faster through the water column by ebullition than by diffusion^40^.

Fluxes in CH_4_ ebullition in inflow water systems are common in the other reservoirs (Table 2), which may be attributable to the fact that water slows down in these areas and sediments have higher chances for deposition^14^. Sediment accumulation rates are positively correlated to the areal organic carbon burial rates^39^, and rapid burial of fresh sediments and organic matter made upstream sites more carbon rich and prime for CH_4_ production by anaerobic metabolism compared to other parts of the reservoirs^12,15,27,37^, as CH_4_ production in reservoirs is strongly driven by organic carbon availability^41^. Thus, ebullitive CH_4_ emissions are often reported to be exponentially increased with corresponding sediment accumulation rates^14,42^. The upstream reaches of Xin’anjiang Reservoir directly receive the catchment and stream inflow of industrial and domestic pollution^43^, which presumably fostered high rates of sediment CH_4_ production in the upstream rivers of the reservoir, causing ebullition zones to subsequently appear^27,29^. Moreover, ebullition rates tend to be highest in shallow areas because short water residence times limit the dissolution of CH_4_-rich bubbles released from the sediment^44^. The upstream river is the shallowest area compared with other regions (see Supplementary Table S3), which is beneficial for bubbles transport from the sediment to the atmosphere because of the small proportion of dissolved gas bubbles during ascent^15,27,37^. Additionally, CH_4_ imported from the Xin’anjiang catchment may further contribute to the observed pattern at river inflow areas.

**Table 2:** Some examples of studies reporting high methane emissions from the upstream inflow areas of reservoir.

location	observations	ref
Three Gorges Reservoir, China	Upstream, reservoir tail waters and tributary sites had higher CH_4_ fluxes than the mainstream of the reservoir.	^3^
Lake Kariba, Zambia/Zimbabwe	Higher fluxes in river deltas (~10^3^ mg CH_4_ m^−2^ d^−1^) than nonriver bay (less than 100 mg CH_4_ m^−2^ d^−1^) due to the high ebullition frequency and ebullition magnitudes.	^27^
Little Nerang Dam, Lake Wivenhoe, Lake Baroon, Australia	CH_4_ saturation was higher in inflow zones than in the main body.	^27^
William H. Harsha Lake, USA	Extreme high CH_4_ emission (mean: 3137 ± 660 mg CH_4_ m^−2^ d^−1^) at the most upstream site; 1 to 2 order of magnitude greater than the other sites.	^27^
Glod Creek Reservoir, Australia	Highest CH_4_ water-air fluxes were found at the main water inflow areas of the reservoir.	^28^
Little Nerang Dam, Australia	1.8–7.0% of the upstream surface area called “ebullition zone”; 97% of the total methane occurred in the ebullition zones.	^29^
Chapéu D’Uvas, Curuá-Una, Furnas, Brazil	Elevated *p*CH_4_ and CH_4_ concentrations in river inflow areas and decreasing values toward the dam; River inflows are hot spots of diffusive C gas flux.	^37^

We recorded higher CH_4_ emissions from the downstream river than from the surface of the reservoir adjacent to the dam (Fig. 2) that had presumably been released from dissolved CH_4_ in the hypolimnion layer of the reservoir^17^ because water inlets of turbines located in the hypolimnion layer (26–37 m under water surface)^43^ and the discharged water derived from the hypolimnion layer almost year round (except February, due to mixing periods). The water adjacent to the dam is thermally stratified, where water in the warmer, upper layer (epilimnion <33 m) is in contact with the atmosphere and is more oxygen-rich, whereas the deeper, colder layer (hypolimnion) contains relatively low levels of O_2_ concentration^25^. We suggest that CH_4_ produced in the reservoir is easily stored in the hypolimnion^45^, and the release of dissolved CH_4_ to the atmosphere occurs due to differences in pressure, temperature, and turbulence when water passes through the turbines and spillways^19^. Water passing through the turbines and spillways is drawn from the hypolimnion, and downstream CH_4_ emissions are released under decreased pressure below the dam^19^.

The explanation for the low CH_4_ emissions from the main reservoir is that the deep, oxic waterbody slows emissions by offering more options for CH_4_ oxidation. Water depths of the sampling points range from 10–69 m, except for those on the margin (Table S3), and it is possible that such reservoir depths increase the possibility of oxidization for diffusive CH_4_ molecules. Moreover, Zhang *et al*. (2015) reported that the DO concentration never fell below 2 mg/L, the critical value for anoxia, in Xin’anjiang Reservoir^25^. The lack of an anoxic layer permits the oxidization of dissolved CH_4_ under aerobic conditions by methanotrophic bacteria^27^. Furthermore, biomass clearing before flooding limited the availability of organic carbon^26,43^, which is important for CH_4_ production in sediments^41^. Chlorophyll a is a significant predictor of CH_4_ emissions from reservoir water surfaces^1,37^, and Xin’anjiang Reservoir is presently in an oligotrophic state, with a low concentration of chlorophyll a (1–3 μg L^−1^)^26^, which limits CH_4_ emissions from the reservoir. Moreover, the dendritic shape of Xin’anjiang Reservoir facilitates the deposition of allochthonous organic carbon in the sediment of the NW lake (see Supplementary Fig. S1)^46^, and limited fresh sediments are deposited in the main reservoir.

### Mitigation strategies for CH_4_ emissions

Management strategies should increase CH_4_ oxidation in the sediments and water columns and decrease CH_4_ production, ebullition, and degassing emission at the dam to mitigate CH_4_ emissions from reservoirs. Extremely allochthonous organic material and organic carbon burial stimulated ebullition in the upstream rivers and river deltas^12,14^; therefore, periodical dredge campaigns^27^, reducing watershed soil erosion^14^ and nutrient input^47^, can efficiently reduce ebullitive CH_4_ emissions. Moreover, the location of spillways and turbines have an impact on CH_4_ emissions from reservoirs^27^.

Previous studies have shown that extreme CH_4_ ebullitive emissions are ultimately attributable to very high sedimentation rates^14^, as well as exhibiting an exponentially increasing relationship between CH_4_ ebullitive emissions and the sediment accumulated rates in the 6 small reservoirs of the Saar River^42^. The mechanism is characterised by deeper sediment layers contributing to CH_4_ formation^42^, and the deeply accumulated CH_4_ causes supersaturation and consequent bubble formation and release^14^. In the study, inflow rivers are ebullition hot spots, thus policymakers should take effective measures to control substantial CH_4_ emissions. The sediment is dredged periodically to reduce deposited organic matter, which presumably decreases the magnitude of ebullitive CH_4_ emissions efficiently, although carbon leakage occurs during the process^27^.

Another practical measure is to prevent the excessive input of nutrients and pollution to the reservoir^30,47,48^, which would reduce the available organic carbon for CH_4_ production^47^. Cage culture is an important nutrient input, which enhances N, P, and TOC accumulation in the sediments of the lacustrine zone^48^. Moreover, the NW lake received more soil erosion, sewage input, and industrial pollution from the upstream rivers in Anhui Province. In response, authorities have taken measures to decrease the inputs of all types of pollution, such as cage culture prohibition and inter-provincial ecological compensation, which improved the water quality in the upstream river from a eutrophic to mesotrophic state, presumably decreasing CH_4_ production and emissions from the reservoir^37^.

Dam design is also important for CH_4_ emissions, especially the location of water intakes. CH_4_ concentrations are higher in the hypolimnion than in the epilimnion during thermal stratification periods^19^. The degassing that occurs as hypolimnion water is routed through a dam accounts for a large fraction (>50%) of the total CH_4_ emissions in some Amazon tropical reservoirs^17,18^. However, if turbine intakes are located in the upper layer of a dam, shallow waters will be withdrawn during thermal stratification to avoid substantial CH_4_ degassing from the CH_4_-rich water in the hypolimnion^19^, for example, only 0.8% from Harsha Lake^27^. Moreover, a significant increase in CH_4_ emissions was reported 3 km upstream from Nam Theum 2 Dam due to the artificial mixing induced by water intakes^33^, and CH_4_-rich water from the reservoir’s hypolimnion reached the surface and resulted in a high CH_4_ diffusive flux. Therefore, the water intake in the hypolimnion not only increased the degassing flux at the dam but also risked enhancing the CH_4_ diffusive flux upstream of the dam.

In summary, upstream rivers are hot spots in bubble CH_4_ emissions, significantly contributing to the total CH_4_ emissions from hydroelectric reservoir systems. If upstream sites are ignored in field-sampling strategies, entire-system CH_4_ emissions will be underestimated. CH_4_ emissions from a main reservoir are lower than that from a downstream river. Capturing the spatial heterogeneity of CH_4_ emissions is vital to estimating the total CH_4_ emissions in a hydroelectric system. Seasonal variation in CH_4_ emissions exhibited a high value in autumn and winter and a low value in spring and summer. A thorough investigation should be conducted for the entire reservoir region over a long period because bubbles are episodic and diffusive CH_4_ emission flux exhibits a strong spatiotemporal variability.

## Materials and Methods

### Study sites

Xin’anjiang Reservoir (118°42′–118°59′E, 29°28′–29°58′N) is located in China’s north subtropical zone. The mean annual air temperature, precipitation, and evaporation are 17.7 °C, 2015.1 mm, and 712.9 mm, respectively (see Supplementary Fig. S2). Constructed in 1959, the reservoir has a water surface area of 580 km^2^ and mean depth of 37 m, with a capacity of approximately 1.78 × 10^10^ m^3^ ^43^, and an annual average inflow and outflow of 9.4 × 10^9^ m^3^ and 9.1 × 10^9^ m^3^, respectively. Water retention time is approximately 2 years, and in 2015, the water level fluctuated between 98 and 104 m above elevation (see Supplementary Fig. S2). According to China’s surface water classification standards, the water quality of Xin’anjiang Reservoir is grade I, serving as an important water source in eastern China that presently provides drinking water.

The reservoir consists of a series of connected lakes in all cardinal directions around a central lake that serves as the main waterbody (see Supplementary Fig. S1). The watercourse of the northwest lake is the dominant source of upstream inflow, contributing 60–80% of the total inflow. The downstream river is the watercourse below Xin’anjiang Dam.

The four sub-lakes and downstream river were sampled at points along transects (see Supplementary Fig. S1). The NW lake transect (118°43′04″E, 29°44′03″N), located in the main upstream inflow inlet, has a width of 0.3 km and three sampling points extending 10, 50, and 120 m (NWP1, NWP2, and NWP3, respectively) from the southern bank marginal zone to the pelagic zone, whereas the NE (119°03′03″E, 29°38′44″N), SW (118°44′39″E, 29°28′18″N), and SE (118°45′20″E, 29°28′39″N) lake transects are located in the open water and have five sampling points (P1 to P5) extending from the marginal to pelagic zones (Table S3). Four sampling points in the downstream river below the dam are located 0.35, 1, 4, and 7 km from Xin’anjiang Dam (DRP1, DRP2, DRP3, and DRP4, respectively).

### CH_4_ flux measurement

Floating static chambers were used to collect gas samples at all sampling points between 08:30 and 11:30 hrs, monthly from December 2014 to December 2015, and bubble traps were used to collect bubbles from the upstream river from August 2016 to November 2017, where samples were collected once or twice per month, except November 2016, and January and February 2017. Air and water temperatures were measured using an alcohol thermometer, and wind speed in the field was measured using an anemometer (Kestrel 1000, Nielsen-Kellerman Co., USA).

Flux of diffusive CH_4_ emissions was collected using floating static chambers and analysed by gas chromatograph. Three floating static chambers (basal area of 0.29 m^2^ and volume of 0.117 m^3^) at each sampling point comprised a non-covered plastic box wrapped in light-reflecting and heatproof materials to minimize internal temperature variation, with plastic foam collars fixed to opposite sides. The headspace height inside the chamber was approximately 35 cm. A silicone tube (0.6 and 0.4 cm outer and inner diameters, respectively) was inserted into the upper central side of the chamber to collect gas samples that were then dried to prevent biological reactions in plexiglass tubes filled with calcium chloride (anhydrous, analytical reagent). Another silicone tube was inserted into the upper corner of the chamber to maintain a balance in air pressure between the inside and outside of the chamber. Static chambers drifted freely behind a boat to reduce measurement bias^49^. Samples of gas were collected from the static chamber in air-sampling bags (0.5 L, Hedetech, Dalian, China) four times every 7 min over a 21-min period using a hand-driven pump (NMP830KNDC, KNF Group, Freiburg, Germany) and were stored until analysis^2^. The air-sampling bags made of aluminium are suitable for gas storage for 7 days and do not absorb or react with CH_4_. Leakage and memory effects of the air-sampling bags were tested in earlier experiments.

We placed 16–26 bubble traps 10–15 m apart in a river crossing rope in the upstream river, where water depth ranged from 5–25 m. The traps consisted of an inverted 30-cm diameter circular funnel fixed to the neck of a 0.56-L plastic bottle, and an additional skirt (50-cm diameter) was fixed to the funnel aperture to enlarge the area over which bubbles were collected^8^. Each funnel was stabilized with three equally sized weights to ensure no tiny bubbles remained in the traps at the initial stage. Trapped gas bubbles liberated from water were collected in the bottles after 24 hours, and then the remaining volume of water was measured to calculate the volume of liberated gas bubbles. The trapped gas was diluted 1000 times by injecting 1 × 10^−3^-L of trapped gas into 1- or 0.5-L gas bags that had been filled with N_2_ to facilitate analysis of CH_4_ concentration by gas chromatography. Trapped gas within these bags was analysed within 3 days using a gas chromatograph (Agilent 7890 A, Agilent Technologies, Santa Clara, USA) equipped with a flame ionization detector (FID). The oven, injector, and detector temperatures were set at 70, 25, and 200 °C, respectively. Standard mixed gas (CH_4_: 1.83 ppm, provided by the China National Research Centre for Certified Reference Materials, Beijing) was used to quantify the CH_4_ concentration in one of every 10 samples, and the coefficient of variation of CH_4_ concentration in the replicated samples was <1%.

The increasing rate of gas concentration (*dc/dt*) within the static chamber was calculated as the slope of the linear regression of the gas concentration versus time. Diffusion chambers collect diffusive emissions as well as ebullitive emissions if they are present. Therefore, if the slope of the linear regression of the gas concentration in the chamber versus time was linear, with R^2^ > 0.9, then the chamber was assumed to collect only diffusive emissions. If R^2^ < 0.9, then the chamber was assumed to collect total (diffusive + ebullitive) emissions^30^.

The flux of diffusive CH_4_ emissions (F_1_, mg CH_4_ m^−2^ h^−1^) is calculated as (Eq. 1):

1 $${F}_{1}=\rho \times \frac{dc}{dt}\times \frac{273.15}{273.15+T}\times H$$

where *ρ* is the density of gas under the standard conditions (0.714 kg m^−3^ for CH_4_), *H* is the height from the top of the inverted chamber to the water surface (here, 0.35 m), 273.15 is the absolute temperature at 0 °C, and T is the air temperate (°C).

The flux of CH_4_ via ebullition (*F*_2_: mg CH_4_ m^−2^ h^−1^), measured as the bubble CH_4_ flux by bubble traps, is calculated as (Eq. 2):

2 $${F}_{2}=\frac{{C}_{CH4}\times V\times M}{{A}_{f}\times t\times {V}_{m}}\times \frac{1}{1000}$$

where *C*_*CH4*_ is the CH_4_ concentration (μL L^−1^), *V* is the accumulated headspace gas volume (L), *M* is the molar weight of CH_4_ (16.04 g mol^−1^), *A*_*f*_ is the funnel area (0.14 m^2^), *t* is the measurement duration (h), and *V*_*m*_ is the molar volume of gas at room temperature (22.4 L mol^−1^)^8^.

The ebullition rate (*ER*; mL m^−2^ h^−1^), which reflects the volume rate of released accumulated bubbles, is calculated as (Eq. 3).

3 $$ER=\frac{V}{{A}_{f}\times t}$$

where the parameters *V*, *A*_*f*_, and *t* are provided in Eq. (2).

### Statistical analysis

The flux in CH_4_ emissions data that did not meet the test for normality (Kolmogorov-Smirnov) were transformed to trigonometric or logarithmic functions prior to testing for seasonal and spatial variability using one-way analysis of variance (ANOVA) and Tukey’s HSD test. Data were analysed using the SPSS statistical package (v. 18.0, Chicago, IL, USA).

## Supplementary information

SI
