# Supplementary material for: Contrasting methane emissions from upstream and downstream rivers and their associated subtropical reservoir in eastern China
Source: Sci Rep. 2019 May 30;9:8072. doi: 10.1038/s41598-019-44470-2 (PMC6542851; doi:10.1038/s41598-019-44470-2)
Supplement: Supplementary file 1 — SI [file 41598_2019_44470_MOESM1_ESM.docx]

Supplementary Information to:

**Contrasting methane emissions from upstream and downstream rivers and their associated subtropical reservoirs in eastern China**

Yang Le

Zhejiang Academy of Forestry, Hangzhou, 310023, China

Corresponding author: E-mail: [yangboshi@live.cn](mailto:yangboshi@live.cn)

**Contents of this file**

Supplementary Equation S1 to S2

Supplementary Figure S1 to S5

Supplementary Table S1 to S9

Average flux in CH_4_ emissions (F_a_; mg CH_4_ m^-2^ h^-1^) from the transects was calculated as (equation S1):

$F_{a}=\frac{\sum_{n=1}^{n\text{=}13} \left[\frac{\sum_{m=1}^{m\text{=}5} (\frac{\sum_{i=1}^{i=3} F_{m}}{i})}{m}\right]}{n}$ (S1)

where, *i* is numbers of chambers; *m* is the number of sampling points within a transect; *n* is the number of times CH_4_ emissions were measured during a given period (Table S.1, S.3-6); and, *F_m_* is CH_4_ emission flux measured by the floating chambers.

Since static floating chambers collect diffusive and bubble CH_4_ emissions, pulses in CH_4_ concentrations were driven by bubbles: therefore, average flux in CH_4_ emissions were calculated as the sum of the frequency of diffusive and ebullitve CH_4_ emissions (*F_a_*) (equation S2):

$F_{a}=f\times F_{ebullition}+\text{（}1\text{-}f\text{）}\times F_{diffusion}$ (S2)

where, *f* is frequency of bubble occurrence; *F_ebullition_* is geometri mean of CH_4_ fluxes in chambers with bubbles; and *F_diffusion_* is the geometric mean of CH_4_ fluxes in bubble free chambers.


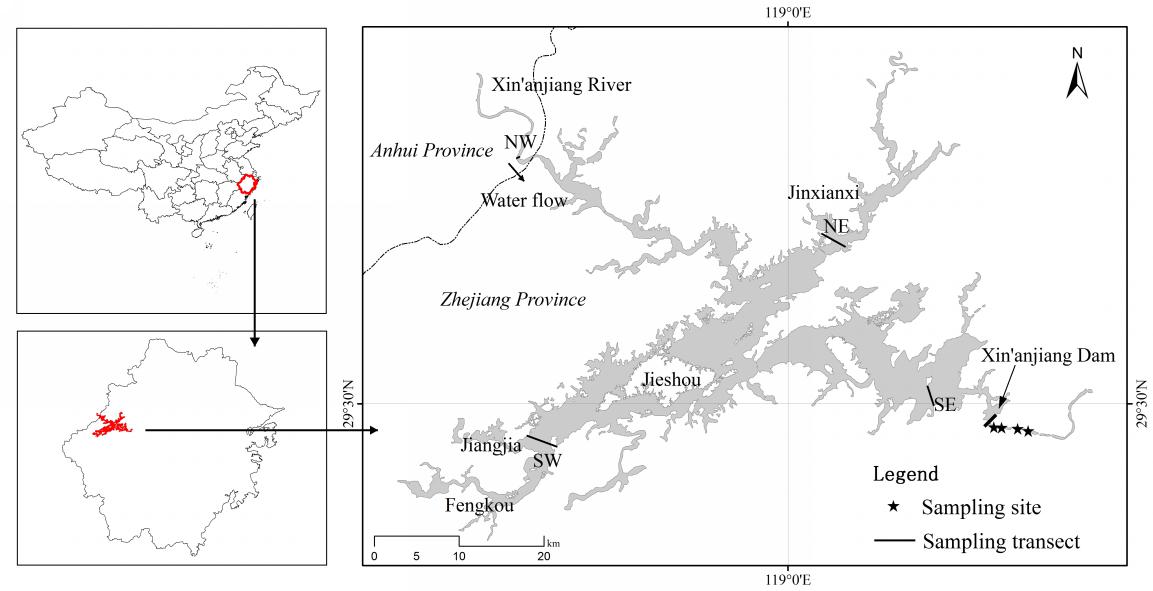


**Supplementary Figure S1**. Location of transects and sampling points.


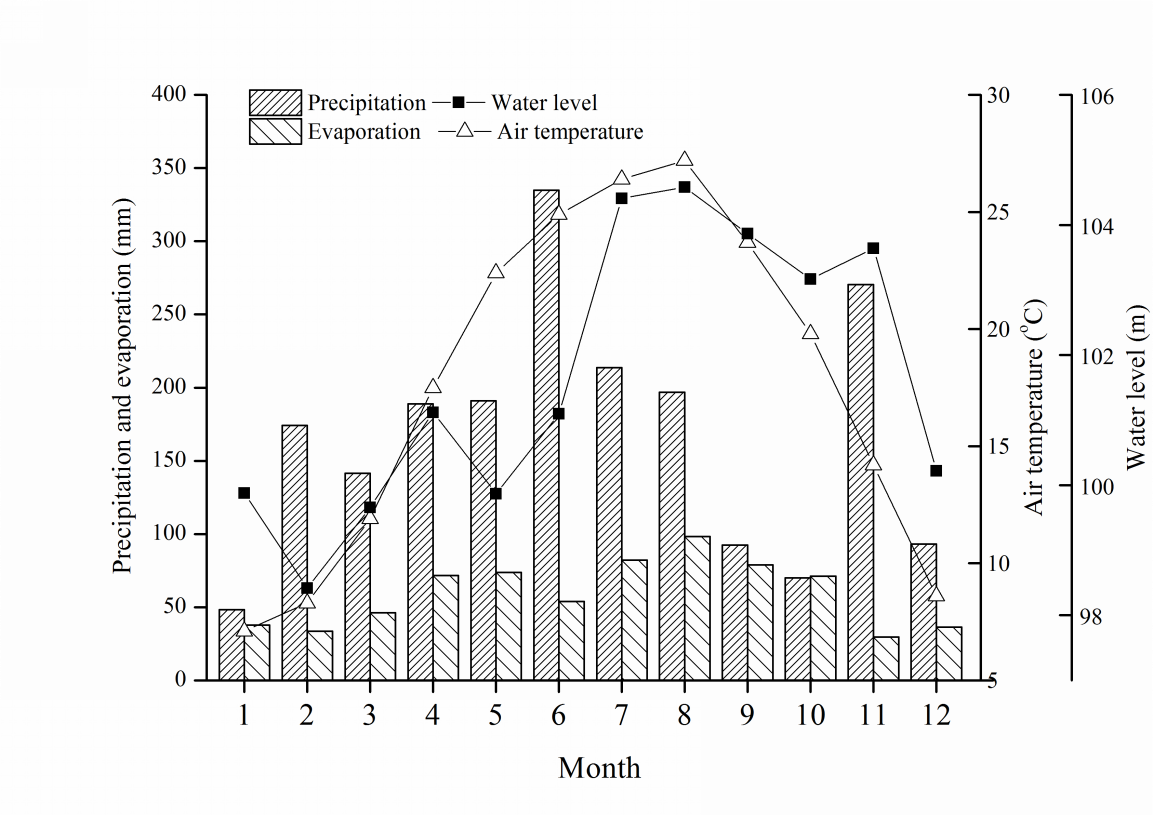


**Supplementary Figure S2.** Monthly precipitation, evaporation, air temperature, and water level in the Xin’anjiang Reservoir in 2015

**Supplementary Figure S3.**  Positive relationships between the ebullitive CH_4_ emission and (a) ebullition rates, (b) bubble CH_4_ concentrations in the NW transect


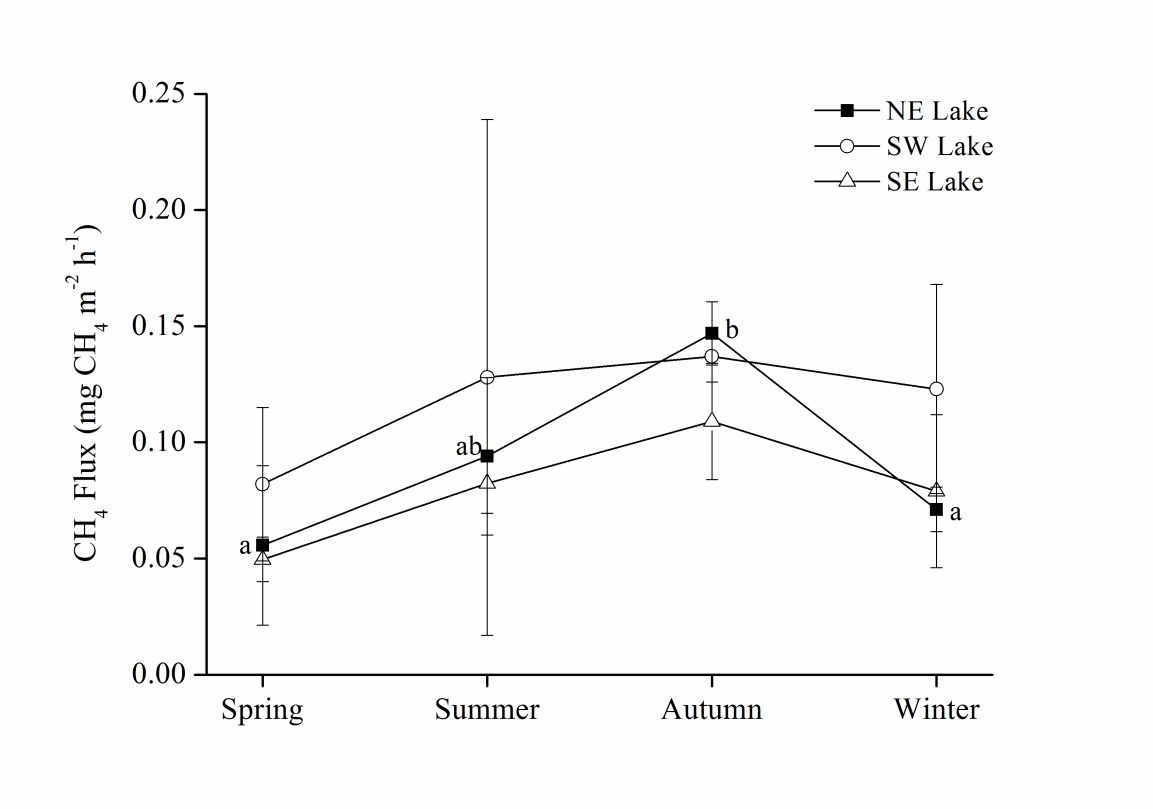


**Supplementary Figure S4.** Seasonal variability in CH_4_ emissions from the three reservoir areas

Note: The different letters marked in Fig. S2 indicated that the significant difference was found in the NE transects among the different seasons


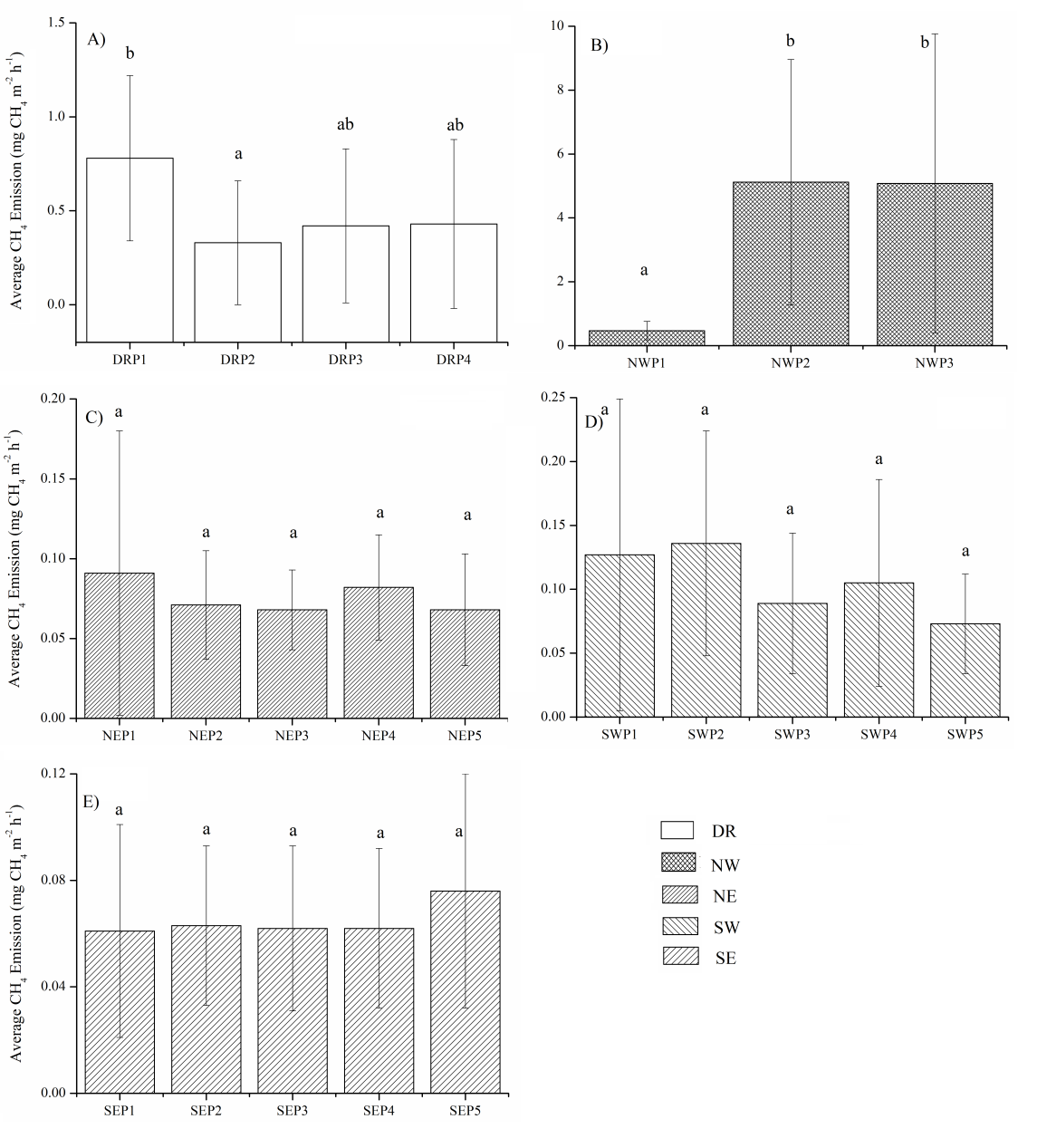


**Supplementary Figure S5.**  Mean CH_4_ emission flux at the each sampling point of different transects

Note: Three to five sampling points (P1 to P5) are located in the lake transects (NW, NE, SW, and SE) and have extending from the marginal to pelagic zones; Four sampling points in the downstream river below the dam are located 0.35, 1, 4, and 7 km from Xin’anjiang Dam (DRP1, DRP2, DRP3, and DRP4, respectively)

**Supplementary Table S1.** Relationship between wind speed and CH_4_ emissions.

| Transect | Equation | *R^2^* | *P-*value | *n* |
| --- | --- | --- | --- | --- |
| NE | y = 0.0058x + 0.051 | 0.063 | 0.047 | 63 |
| SE | y = 0.0060x + 0.047 | 0.097 | 0.010 | 67 |
| SW | y = 0.017x + 0.047 | 0.19 | 0.000 | 64 |

**Supplementary Table S2.** Relationships between the difference of air-water temperature and CH_4_ emissions.

| Transect | Equation | *R^2^* | *P*-value | *n* |
| --- | --- | --- | --- | --- |
| DR | y = 0.034x + 0.51 | 0.29 | 0.000 | 56 |
| NW | y = 0.31x + 3.15 | 0.15 | 0.014 | 39 |
| SE | y = 0.002x + 0.065 | 0.059 | 0.047 | 67 |
| SW | y = 0.012x + 0.056 | 0.17 | 0.001 | 62 |
| NE | y = 0.0023x + 0.070 | 0.042 | 0.105 | 63 |

**Supplementary Table 3. Water depths of the 22 sampling points at the water level of 102 m at the five transects.**

| Transect | Sampling Point | | | | |
| --- | --- | --- | --- | --- | --- |
|  | P1 | P2 | P3 | P4 | P5 |
| NW | 5 | 23 | 20 |  |  |
| NE | 5 | 11 | 35 | 39 | 45 |
| SW | 4 | 10 | 26 | 30 | 26 |
| SE | 6 | 24 | 60 | 69 | 55 |
| DR | 5 | 6 | 6 | 6 |  |

**Supplementary Table 4.** Complete dataset of the measured CH_4_ emission fluxes by the floating chambers at the 3 sampling points of NW transect from Dec. 2014 to Jan. 2016.

| Dates | Sampling points | Chamber 1 | Chamber 2 | Chamber 3 |
| --- | --- | --- | --- | --- |
| 20141214 | NWD1 | 0.78 | 0.95 | 0.76 |
| 20141214 | NWD2 | 0.27 | 10.51^*^ | 0.17 |
| 20141214 | NWD3 | 37.70^*^ | 0.47 | 0.33 |
| 20150109 | NWD1 | 0.44 | 0.36 | 0.45 |
| 20150109 | NWD2 | 0.53 | 35.07^*^ | 1.33^*^ |
| 20150109 | NWD3 | 0.37 | 10.38^*^ | 30.47^*^ |
| 20150204 | NWD1 | 0.23 | 0.27 | 0.57 |
| 20150204 | NWD2 | 0.27 | 0.24 | 0.32 |
| 20150204 | NWD3 | 0.42 | 1.94^*^ | 3.18^*^ |
| 20150312 | NWD1 | 0.038 | No data | No data |
| 20150312 | NWD2 | 0.018 | No data | 0.035 |
| 20150312 | NWD3 | 0.24 | No data | 0.008 |
| 20150418 | NWD1 | 0.37 | 0.28 | 0.50 |
| 20150418 | NWD2 | 0.35 | 0.23 | 0.26 |
| 20150418 | NWD3 | 0.48 | 0.78 | 1.15 |
| 20150520 | NWD1 | 1.10 | 0.91 | 0.55 |
| 20150520 | NWD2 | 1.15 | 0.79 | 7.15^*^ |
| 20150520 | NWD3 | 2.81 | 3.01 | 3.07 |
| 20150624 | NWD1 | 0.89 | 0.97 | 0.92 |
| 20150624 | NWD2 | 0.97 | 0.75 | 0.99 |
| 20150624 | NWD3 | 0.50 | 0.93 | 4.07^*^ |
| 20150725 | NWD1 | 0.84 | 0.75 | 0.48 |
| 20150725 | NWD2 | 7.70^*^ | 2.73 | 17.06^*^ |
| 20150725 | NWD3 | 1.01 | 0.82 | 1.06 |
| 20150819 | NWD1 | 0.98 | 0.43 | 0.49 |
| 20150819 | NWD2 | 0.68 | 0.31 | 0.40 |
| 20150819 | NWD3 | 0.94 | 0.88 | 0.81 |
| 20150921 | NWD1 | 0.22 | 0.30 | 0.37 |
| 20150921 | NWD2 | 5.88 | 33.62^*^ | 6.01 |
| 20150921 | NWD3 | 4.81 | 5.31 | 7.45 |
| 20151015 | NWD1 | 0.13 | 0.019 | 0.31 |
| 20151015 | NWD2 | 3.91 | 3.74 | 2.15 |
| 20151015 | NWD3 | 2.36 | 6.04 | 28.53^*^ |
| 20151127 | NWD1 | 0.42 | 0.33 | 0.53 |
| 20151127 | NWD2 | 18.06^*^ | 0.47 | 0.36 |
| 20151127 | NWD3 | 0.31 | 0.15 | 0.24 |
| 20160109 | NWD1 | 0.095 | 0.14 | No data |
| 20160109 | NWD2 | 1.63 | 27.57^*^ | 0.998 |
| 20160109 | NWD3 | 1.44 | 20.50^*^ | 8.18^*^ |

Note: The unit of CH_4_ flux is mg CH_4_ m^-2^ h^-1^. NWP1, NWP2, and NWP3 are the 3 fixed sampling points in the NW transect. The sign of star (*) means that the bubbles were trapped in a chamber during the gas collected periods (21 minutes), which was judged by whether the CH4 concentrations increased abruptly in the floating chambers.

**Supplementary Table S5.** Complete dataset of the measured ebullitive CH4 fluxs, ebullition rates, and CH4 concentrations by the inverted funnels in the 26 sampling stations of the NW transect during Aug. 2016 to Nov. 2017

| Dates | Loca-tions | Trapped gas volume (ml) | Deployment time (h) | F  (mg CH_4_ m^-2^ h^-1^) | ER  (ml m^-2^ h^-1^ ) | C (%) |
| --- | --- | --- | --- | --- | --- | --- |
| 20160803 | 1 | 165 | 19.9 | 21.97 | 59.28 | 51.75 |
| 20160803 | 2 | 125 | 20 | 16.88 | 44.64 | 52.80 |
| 20160803 | 3 | 90 | 20.1 | 11.96 | 31.95 | 52.26 |
| 20160803 | 4 | 40 | 20.2 | 3.77 | 14.12 | 37.31 |
| 20160803 | 5 | 30 | 20.4 | 2.06 | 10.52 | 27.40 |
| 20160803 | 6 | 10 | 20.5 | 0.52 | 3.49 | 20.76 |
| 20160803 | 7 | 50 | 20.6 | 4.11 | 17.34 | 33.14 |
| 20160803 | 8 | 15 | 20.7 | 1.12 | 5.17 | 30.26 |
| 20160803 | 9 | 75 | 20.8 | 9.18 | 25.71 | 49.85 |
| 20160803 | 10 | 150 | 21 | 19.07 | 51.12 | 52.10 |
| 20160803 | 11 | 45 | 21.1 | 5.13 | 15.25 | 47.03 |
| 20160803 | 12 | 140 | 21.2 | 23.52 | 47.17 | 69.62 |
| 20160803 | 13 | 145 | 21.3 | 19.99 | 48.58 | 57.45 |
| 20160803 | 14 | 35 | 21.4 | 3.82 | 11.66 | 45.77 |
| 20160803 | 15 | 105 | 21.6 | 13.29 | 34.79 | 53.36 |
| 20160803 | 16 | 560 | 21.7 | 93.06 | 184.50 | 70.44 |
| 20160803 | 17 | 20 | 21.8 | 1.79 | 6.55 | 38.19 |
| 20160803 | 18 | 110 | 21.9 | 13.92 | 35.84 | 54.24 |
| 20160803 | 19 | 110 | 22 | 19.22 | 35.65 | 75.30 |
| 20160803 | 20 | 135 | 22.2 | 19.43 | 43.51 | 62.34 |
| 20160803 | 21 | 210 | 22.3 | 33.35 | 67.32 | 69.17 |
| 20160803 | 22 | 150 | 22.4 | 20.44 | 47.83 | 59.67 |
| 20160803 | 23 | 70 | 22.5 | 10.84 | 22.20 | 68.21 |
| 20160803 | 24 | 210 | 22.6 | 25.72 | 66.25 | 54.20 |
| 20160803 | 25 | 0 | 22.8 | 0 | 0 | 0 |
| 20160803 | 26 | 0 | 22.9 | 0 | 0 | 0 |
| 20160818 | 1 | 130 | 21.5 | 13.74 | 43.18 | 44.43 |
| 20160818 | 2 | 90 | 21.7 | 10.77 | 29.67 | 50.72 |
| 20160818 | 3 | 90 | 21.8 | 10.51 | 29.55 | 49.66 |
| 20160818 | 4 | 90 | 21.8 | 11.27 | 29.44 | 53.45 |
| 20160818 | 5 | 374 | 21.9 | 49.51 | 121.88 | 56.73 |
| 20160818 | 6 | 170 | 22.0 | 24.90 | 55.19 | 63.01 |
| 20160818 | 7 | 190 | 22.1 | 26.97 | 61.45 | 61.29 |
| 20160818 | 8 | 105 | 22.2 | 14.29 | 33.83 | 58.99 |
| 20160818 | 9 | 125 | 22.3 | 20.74 | 40.13 | 72.19 |
| 20160818 | 10 | 315 | 22.3 | 51.97 | 100.74 | 72.05 |
| 20160818 | 11 | 280 | 22.4 | 39.86 | 89.22 | 62.40 |
| 20160818 | 12 | 85 | 22.5 | 12.50 | 26.98 | 64.66 |
| 20160818 | 13 | 235 | 22.6 | 42.39 | 74.33 | 79.64 |
| 20160818 | 14 | 240 | 22.7 | 37.31 | 75.63 | 68.89 |
| 20160818 | 15 | 100 | 22.7 | 14.70 | 31.40 | 65.38 |
| 20160818 | 16 | 290 | 22.8 | 39.60 | 90.72 | 60.95 |
| 20160818 | 17 | 335 | 22.9 | 53.49 | 104.42 | 71.53 |
| 20160818 | 18 | 300 | 23.0 | 47.24 | 93.18 | 70.80 |
| 20160818 | 19 | 432 | 23.1 | 63.22 | 133.69 | 66.04 |
| 20160818 | 20 | 428 | 23.2 | 64.70 | 131.98 | 68.46 |
| 20160818 | 21 | 300 | 23.2 | 37.93 | 92.18 | 57.46 |
| 20160818 | 22 | 0 | 23.3 | 0 | 0 | 0 |
| 20160911 | 1 | 290 | 22 | 41.39 | 94.32 | 61.28 |
| 20160911 | 2 | 650 | 22 | 103.16 | 210.60 | 68.41 |
| 20160911 | 3 | 220 | 22.1 | 30.82 | 71.01 | 60.62 |
| 20160911 | 4 | 90 | 22.2 | 10.17 | 28.94 | 49.06 |
| 20160911 | 5 | 120 | 22.3 | 15.62 | 38.43 | 56.77 |
| 20160911 | 6 | 70 | 22.4 | 7.58 | 22.33 | 47.38 |
| 20160911 | 7 | 90 | 22.5 | 11.16 | 28.61 | 54.50 |
| 20160911 | 8 | 70 | 22.6 | 9.60 | 22.17 | 60.47 |
| 20160911 | 9 | 175 | 22.6 | 29.93 | 55.21 | 75.72 |
| 20160911 | 10 | 85 | 22.7 | 11.60 | 26.71 | 60.64 |
| 20160911 | 11 | 295 | 22.8 | 46.06 | 92.37 | 69.64 |
| 20160911 | 12 | 210 | 22.9 | 31.45 | 65.51 | 67.04 |
| 20160911 | 13 | 275 | 23.0 | 42.49 | 85.47 | 69.42 |
| 20160911 | 14 | 225 | 23.1 | 39.74 | 69.67 | 79.67 |
| 20160911 | 15 | 700 | 23.2 | 114.27 | 215.95 | 73.90 |
| 20160911 | 16 | 135 | 23.2 | 21.89 | 41.49 | 73.66 |
| 20160911 | 17 | 280 | 23.3 | 44.48 | 85.75 | 72.43 |
| 20160911 | 18 | 295 | 23.4 | 39.19 | 90.01 | 60.80 |
| 20160911 | 19 | 330 | 23.5 | 58.32 | 100.33 | 81.18 |
| 20160911 | 20 | 366 | 23.6 | 50.75 | 110.87 | 63.92 |
| 20160911 | 21 | 135 | 23.7 | 18.47 | 40.75 | 63.31 |
| 20160911 | 22 | 0 | 23.75 | 0 | 0 | 0 |
| 20160912 | 1 | 85 | 18 | 14.75 | 33.73 | 61.08 |
| 20160912 | 2 | 215 | 18 | 37.64 | 85.32 | 61.62 |
| 20160912 | 3 | 20 | 18 | 3.68 | 7.94 | 64.83 |
| 20160912 | 4 | 240 | 18 | 52.08 | 95.24 | 76.37 |
| 20161010 | 1 | 380 | 22.1 | 71.84 | 123.01 | 81.52 |
| 20161010 | 2 | 195 | 22.1 | 35.62 | 62.94 | 79.01 |
| 20161010 | 3 | 245 | 22.2 | 45.92 | 78.85 | 81.30 |
| 20161010 | 4 | 260 | 22.3 | 44.71 | 83.43 | 74.81 |
| 20161010 | 5 | 290 | 22.3 | 48.48 | 92.79 | 72.94 |
| 20161010 | 6 | 245 | 22.4 | 40.74 | 78.16 | 72.77 |
| 20161010 | 7 | 70 | 22.5 | 10.81 | 22.27 | 67.76 |
| 20161010 | 8 | 140 | 22.5 | 24.28 | 44.40 | 76.33 |
| 20161010 | 9 | 110 | 22.6 | 18.88 | 34.79 | 75.75 |
| 20161010 | 10 | 70 | 22.7 | 11.86 | 22.08 | 74.99 |
| 20161010 | 11 | 155 | 22.7 | 28.57 | 48.74 | 81.83 |
| 20161010 | 12 | 110 | 22.8 | 20.67 | 34.49 | 83.67 |
| 20161010 | 13 | 120 | 22.8 | 22.76 | 37.52 | 84.66 |
| 20161010 | 14 | 750 | 22.9 | 149.72 | 233.83 | 89.38 |
| 20161010 | 15 | 155 | 23 | 30.50 | 48.19 | 88.35 |
| 20161010 | 16 | 250 | 23 | 47.59 | 77.50 | 85.72 |
| 20161010 | 17 | 205 | 23.1 | 38.19 | 63.38 | 84.11 |
| 20161010 | 18 | 110 | 23.2 | 17.69 | 33.91 | 72.82 |
| 20161010 | 19 | 90 | 23.2 | 12.00 | 27.67 | 60.53 |
| 20161010 | 20 | 0 | 23.3 | 0 | 0 | 0 |
| 20161206 | 1 | 112 | 20.9 | 12.33 | 38.34 | 44.90 |
| 20161206 | 2 | 300 | 20.9 | 50.19 | 102.50 | 68.38 |
| 20161206 | 3 | 76 | 20.9 | 11.93 | 25.92 | 64.30 |
| 20161206 | 4 | 72 | 21 | 12.27 | 24.51 | 69.92 |
| 20161206 | 5 | 40 | 21 | 6.23 | 13.59 | 63.98 |
| 20161206 | 6 | 20 | 21.1 | 2.33 | 6.78 | 48.05 |
| 20161206 | 7 | 30 | 21.1 | 2.50 | 10.14 | 34.41 |
| 20161206 | 8 | 0 | 21.2 | 0 | 0 | 0 |
| 20161206 | 9 | 140 | 21.2 | 24.07 | 47.14 | 71.32 |
| 20161206 | 10 | 0 | 21.3 | 0 | 0 | 0 |
| 20161206 | 11 | 73 | 21.3 | 12.53 | 24.49 | 71.44 |
| 20161206 | 12 | 0 | 21.3 | 0 | 0 | 0 |
| 20161206 | 13 | 20 | 21.4 | 2.26 | 6.69 | 47.24 |
| 20161206 | 14 | 15 | 21.4 | 1.39 | 5.01 | 38.69 |
| 20161206 | 15 | 0 | 21.4 | 0 | 0 | 0 |
| 20161206 | 16 | 40 | 21.5 | 6.34 | 13.30 | 66.57 |
| 20161206 | 17 | 35 | 21.5 | 5.30 | 11.62 | 63.70 |
| 20161206 | 18 | 55 | 21.6 | 7.66 | 18.22 | 58.66 |
| 20161206 | 19 | 35 | 21.6 | 3.93 | 11.58 | 47.44 |
| 20161206 | 20 | 30 | 21.6 | 4.12 | 9.90 | 58.03 |
| 20161206 | 21 | 100 | 21.7 | 15.66 | 32.96 | 66.36 |
| 20161206 | 22 | 55 | 21.7 | 8.22 | 18.09 | 63.40 |
| 20161206 | 23 | 0 | 21.7 | 0 | 0 | 0 |
| 20170325 | 1 | 0 | 20.8 | 0 | 0 | 0 |
| 20170325 | 2 | 0 | 20.9 | 0 | 0 | 0 |
| 20170325 | 3 | 0 | 20.9 | 0 | 0 | 0 |
| 20170325 | 4 | 0 | 20.9 | 0 | 0 | 0 |
| 20170325 | 5 | 10 | 21 | 0.92 | 3.40 | 37.69 |
| 20170325 | 6 | 0 | 21 | 0 | 0 | 0 |
| 20170325 | 7 | 0 | 21.1 | 0 | 0 | 0 |
| 20170325 | 8 | 0 | 21.1 | 0 | 0 | 0 |
| 20170325 | 9 | 15 | 21.1 | 1.97 | 5.07 | 54.19 |
| 20170325 | 10 | 0 | 21.2 | 0 | 0 | 0 |
| 20170325 | 11 | 0 | 21.2 | 0 | 0 | 0 |
| 20170325 | 12 | 0 | 21.3 | 0 | 0 | 0 |
| 20170325 | 13 | 0 | 21.3 | 0 | 0 | 0 |
| 20170325 | 14 | 0 | 21.3 | 0 | 0 | 0 |
| 20170325 | 15 | 0 | 21.4 | 0 | 0 | 0 |
| 20170325 | 16 | 0 | 21.4 | 0 | 0 | 0 |
| 20170325 | 17 | 0 | 21.4 | 0 | 0 | 0 |
| 20170325 | 18 | 45 | 21.5 | 5.85 | 14.96 | 54.56 |
| 20170325 | 19 | 0 | 21.5 | 0 | 0 | 0 |
| 20170325 | 20 | 0 | 21.6 | 0 | 0 | 0 |
| 20170412 | 1 | 0 | 20.8 | 0 | 0 | 0 |
| 20170412 | 2 | 20 | 31.5 | 0.30 | 4.54 | 9.23 |
| 20170412 | 3 | 0 | 31.5 | 0 | 0 | 0 |
| 20170412 | 4 | 0 | 31.5 | 0 | 0 | 0 |
| 20170412 | 5 | 0 | 31.5 | 0 | 0 | 0 |
| 20170412 | 6 | 0 | 31.5 | 0 | 0 | 0 |
| 20170412 | 7 | 20 | 31.5 | 1.77 | 4.54 | 54.57 |
| 20170412 | 8 | 0 | 31.5 | 0 | 0 | 0 |
| 20170412 | 9 | 0 | 31.5 | 0 | 0 | 0 |
| 20170412 | 10 | 15 | 31.5 | 0.43 | 3.40 | 17.80 |
| 20170412 | 11 | 0 | 31.5 | 0 | 0 | 0 |
| 20170412 | 12 | 0 | 31.5 | 0 | 0 | 0 |
| 20170412 | 13 | 5 | 31.5 | 0.39 | 1.13 | 47.65 |
| 20170412 | 14 | 20 | 31.5 | 1.35 | 4.54 | 41.54 |
| 20170412 | 15 | 10 | 31.5 | 0.63 | 2.27 | 38.94 |
| 20170412 | 16 | 0 | 31.5 | 0 | 0 | 0 |
| 20170427 | 1 | 0 | 20.8 | 0 | 0 | 0 |
| 20170427 | 2 | 0 | 32.5 | 0 | 0 | 0 |
| 20170427 | 3 | 10 | 32.5 | 0.64 | 2.20 | 40.93 |
| 20170427 | 4 | 20 | 32.5 | 1.83 | 4.40 | 58.16 |
| 20170427 | 5 | 0 | 32.5 | 0 | 0 | 0 |
| 20170427 | 6 | 50 | 32.5 | 5.58 | 10.99 | 70.97 |
| 20170427 | 7 | 50 | 32.5 | 5.99 | 10.99 | 76.19 |
| 20170427 | 8 | 0 | 32.5 | 0 | 0 | 0 |
| 20170427 | 9 | 0 | 32.5 | 0 | 0 | 0 |
| 20170427 | 10 | 20 | 32.5 | 2.19 | 4.40 | 69.46 |
| 20170427 | 11 | 60 | 32.5 | 8.28 | 13.19 | 87.69 |
| 20170427 | 12 | 50 | 32.5 | 6.16 | 10.99 | 78.33 |
| 20170427 | 13 | 110 | 32.5 | 14.70 | 24.18 | 84.91 |
| 20170427 | 14 | 150 | 32.5 | 20.72 | 32.97 | 87.79 |
| 20170427 | 15 | 70 | 32.5 | 8.94 | 15.38 | 81.15 |
| 20170427 | 16 | 60 | 32.5 | 7.30 | 13.19 | 77.26 |
| 20170512 | 1 | 0 | 24 | 0 | 0 | 0 |
| 20170512 | 2 | 30 | 24 | 3.72 | 8.93 | 58.15 |
| 20170512 | 3 | 10 | 24 | 1.02 | 2.98 | 47.74 |
| 20170512 | 4 | 0 | 24 | 0 | 0 | 0 |
| 20170512 | 5 | 0 | 24 | 0 | 0 | 0 |
| 20170512 | 6 | 5 | 24 | 0.44 | 1.49 | 41.42 |
| 20170512 | 7 | 50 | 24 | 7.81 | 14.88 | 73.25 |
| 20170512 | 8 | 0 | 24 | 0 | 0 | 0 |
| 20170512 | 9 | 0 | 24 | 0 | 0 | 0 |
| 20170512 | 10 | 0 | 24 | 0 | 0 | 0 |
| 20170512 | 11 | 0 | 24 | 0 | 0 | 0 |
| 20170512 | 12 | 170 | 24 | 31.47 | 50.60 | 86.86 |
| 20170512 | 13 | 50 | 24 | 9.30 | 14.88 | 87.21 |
| 20170512 | 14 | 80 | 24 | 14.94 | 23.81 | 87.63 |
| 20170512 | 15 | 170 | 24 | 31.00 | 50.60 | 85.56 |
| 20170512 | 16 | 60 | 24 | 9.49 | 17.86 | 74.19 |
| 20170512 | 17 | 20 | 24 | 2.88 | 5.95 | 67.50 |
| 20170527 | 1 | 225 | 24 | 28.83 | 66.96 | 60.12 |
| 20170527 | 2 | 185 | 24 | 28.74 | 55.06 | 72.90 |
| 20170527 | 3 | 145 | 24 | 22.08 | 43.15 | 71.45 |
| 20170527 | 4 | 50 | 24 | 6.32 | 14.88 | 59.35 |
| 20170527 | 5 | 130 | 24 | 18.76 | 38.69 | 67.71 |
| 20170527 | 6 | 330 | 24 | 59.57 | 98.21 | 84.70 |
| 20170527 | 7 | 120 | 24 | 15.83 | 35.71 | 61.89 |
| 20170527 | 8 | 40 | 24 | 4.60 | 11.90 | 53.91 |
| 20170527 | 9 | 70 | 24 | 9.12 | 20.83 | 61.15 |
| 20170527 | 10 | 90 | 24 | 13.00 | 26.79 | 67.75 |
| 20170527 | 11 | 80 | 24 | 13.08 | 23.81 | 76.75 |
| 20170527 | 12 | 70 | 24 | 10.81 | 20.83 | 72.48 |
| 20170527 | 13 | 100 | 24 | 15.91 | 29.76 | 74.65 |
| 20170527 | 14 | 90 | 24 | 14.97 | 26.79 | 78.04 |
| 20170527 | 15 | 340 | 24 | 60.77 | 101.19 | 83.86 |
| 20170527 | 16 | 290 | 24 | 50.48 | 86.31 | 81.68 |
| 20170527 | 17 | 130 | 24 | 21.18 | 38.69 | 77.52 |
| 20170527 | 18 | 90 | 24 | 15.18 | 26.79 | 79.12 |
| 20170527 | 19 | 110 | 24 | 18.17 | 32.74 | 77.51 |
| 20170527 | 20 | 0 | 24 | 0 | 0 | 0 |
| 20170603 | 1 | 215 | 24 | 30.77 | 63.99 | 67.15 |
| 20170603 | 2 | 195 | 24 | 30.47 | 58.04 | 73.31 |
| 20170603 | 3 | 160 | 24 | 25.13 | 47.62 | 73.70 |
| 20170603 | 4 | 80 | 24 | 12.42 | 23.81 | 72.83 |
| 20170603 | 5 | 150 | 24 | 24.16 | 44.64 | 75.57 |
| 20170603 | 6 | 90 | 24 | 13.23 | 26.79 | 68.95 |
| 20170603 | 7 | 90 | 24 | 10.50 | 26.79 | 54.72 |
| 20170603 | 8 | 120 | 24 | 16.57 | 35.71 | 64.78 |
| 20170603 | 9 | 20 | 24 | 1.90 | 5.95 | 44.48 |
| 20170603 | 10 | 30 | 24 | 3.09 | 8.93 | 48.31 |
| 20170603 | 11 | 550 | 24 | 90.48 | 163.69 | 77.19 |
| 20170603 | 12 | 20 | 24 | 1.94 | 5.95 | 45.70 |
| 20170603 | 13 | 20 | 24 | 3.13 | 5.95 | 73.36 |
| 20170603 | 14 | 50 | 24 | 7.58 | 14.88 | 71.13 |
| 20170603 | 15 | 170 | 24 | 32.38 | 50.60 | 89.37 |
| 20170603 | 16 | 270 | 24 | 53.09 | 80.36 | 92.27 |
| 20170603 | 17 | 490 | 24 | 92.86 | 145.83 | 88.92 |
| 20170603 | 18 | 160 | 24 | 30.51 | 47.62 | 89.47 |
| 20170603 | 19 | 130 | 24 | 19.94 | 38.69 | 71.98 |
| 20170603 | 20 | 150 | 24 | 28.56 | 44.64 | 89.34 |
| 20170603 | 21 | 230 | 24 | 42.90 | 68.45 | 87.53 |
| 20170603 | 22 | 120 | 24 | 17.91 | 35.71 | 70.02 |
| 20170603 | 23 | 510 | 24 | 92.21 | 151.79 | 84.84 |
| 20170603 | 24 | 160 | 24 | 28.44 | 47.62 | 83.41 |
| 20170603 | 25 | 0 | 24 | 0 | 0 | 0 |
| 20170712 | 1 | 40 | 24 | 3.27 | 11.70 | 38.36 |
| 20170712 | 2 | 30 | 24 | 2.54 | 8.93 | 39.80 |
| 20170712 | 3 | 530 | 24 | 73.99 | 157.74 | 65.51 |
| 20170712 | 4 | 0 | 24 | 0 | 0 | 0 |
| 20170712 | 5 | 0 | 24 | 0 | 0 | 0 |
| 20170712 | 6 | 0 | 24 | 0 | 0 | 0 |
| 20170712 | 7 | 0 | 24 | 0 | 0 | 0 |
| 20170712 | 8 | 20 | 24 | 2.36 | 5.95 | 55.36 |
| 20170712 | 9 | 0 | 24 | 0 | 0 | 0 |
| 20170712 | 10 | 0 | 24 | 0 | 0 | 0 |
| 20170712 | 11 | 30 | 24 | 3.30 | 8.93 | 51.68 |
| 20170712 | 12 | 20 | 24 | 1.93 | 5.95 | 45.11 |
| 20170712 | 13 | 30 | 24 | 2.85 | 8.92 | 44.63 |
| 20170712 | 14 | 60 | 24 | 6.73 | 17.86 | 52.67 |
| 20170712 | 15 | 20 | 24 | 1.93 | 5.95 | 45.25 |
| 20170712 | 16 | 0 | 24 | 0 | 0 | 0 |
| 20170712 | 17 | 0 | 24 | 0 | 0 | 0 |
| 20170712 | 18 | 20 | 24 | 1.73 | 5.95 | 40.49 |
| 20170712 | 19 | 0 | 24 | 0 | 0 | 0 |
| 20170712 | 20 | 60 | 24 | 6.31 | 17.86 | 49.35 |
| 20170712 | 21 | 40 | 24 | 3.69 | 11.90 | 43.27 |
| 20170712 | 22 | 0 | 24 | 0 | 0 | 0 |
| 20170712 | 23 | 30 | 24 | 2.74 | 8.93 | 42.81 |
| 20170712 | 24 | 0 | 24 | 0 | 0 | 0 |
| 20170712 | 25 | 20 | 24 | 1.58 | 5.95 | 36.99 |
| 20170712 | 26 | 0 | 24 | 0 | 0 | 0 |
| 20170725 | 1 | 150 | 24 | 13.98 | 44.64 | 43.75 |
| 20170725 | 2 | 550 | 24 | 75.78 | 163.69 | 64.65 |
| 20170725 | 3 | 70 | 24 | 8.79 | 20.83 | 58.92 |
| 20170725 | 4 | 90 | 24 | 11.54 | 26.79 | 60.15 |
| 20170725 | 5 | 90 | 24 | 11.26 | 26.79 | 58.69 |
| 20170725 | 6 | 50 | 24 | 6.71 | 14.88 | 62.96 |
| 20170725 | 7 | 50 | 24 | 9.93 | 14.88 | 93.20 |
| 20170725 | 8 | 40 | 24 | 4.99 | 11.90 | 58.56 |
| 20170725 | 9 | 160 | 24 | 22.57 | 47.62 | 66.20 |
| 20170725 | 10 | 170 | 24 | 29.72 | 50.60 | 82.04 |
| 20170725 | 11 | 50 | 24 | 7.71 | 14.88 | 72.35 |
| 20170725 | 12 | 60 | 24 | 9.59 | 17.86 | 75.00 |
| 20170725 | 13 | 220 | 24 | 40.13 | 65.48 | 85.59 |
| 20170725 | 14 | 120 | 24 | 21.27 | 35.71 | 83.18 |
| 20170725 | 15 | 80 | 24 | 13.27 | 23.81 | 77.84 |
| 20170725 | 16 | 240 | 24 | 44.11 | 71.43 | 86.23 |
| 20170725 | 17 | 60 | 24 | 10.60 | 17.86 | 82.88 |
| 20170725 | 18 | 210 | 24 | 35.91 | 62.50 | 80.24 |
| 20170725 | 19 | 230 | 24 | 41.23 | 68.45 | 84.11 |
| 20170725 | 20 | 170 | 24 | 29.82 | 50.60 | 82.31 |
| 20170725 | 21 | 180 | 24 | 30.82 | 53.57 | 80.35 |
| 20170725 | 22 | 120 | 24 | 19.92 | 35.71 | 77.88 |
| 20170725 | 23 | 70 | 24 | 11.27 | 20.83 | 75.52 |
| 20170725 | 24 | 60 | 24 | 8.75 | 17.86 | 68.46 |
| 20170725 | 25 | 0 | 24 | 0 | 0 | 0 |
| 20170803 | 1 | 310 | 24 | 46.81 | 92.26 | 70.86 |
| 20170803 | 2 | 150 | 24 | 19.71 | 44.64 | 61.64 |
| 20170803 | 3 | 210 | 24 | 31.58 | 62.50 | 70.55 |
| 20170803 | 4 | 90 | 24 | 12.21 | 26.79 | 63.66 |
| 20170803 | 5 | 90 | 24 | 12.94 | 26.79 | 67.46 |
| 20170803 | 6 | 80 | 24 | 11.87 | 23.81 | 69.64 |
| 20170803 | 7 | 130 | 24 | 20.73 | 38.69 | 74.83 |
| 20170803 | 8 | 100 | 24 | 15.55 | 29.76 | 72.94 |
| 20170803 | 9 | 140 | 24 | 24.32 | 41.67 | 81.52 |
| 20170803 | 10 | 140 | 24 | 23.48 | 41.67 | 78.70 |
| 20170803 | 11 | 250 | 24 | 47.01 | 74.40 | 88.24 |
| 20170803 | 12 | 110 | 24 | 19.29 | 32.74 | 82.30 |
| 20170803 | 13 | 110 | 24 | 19.89 | 32.74 | 84.85 |
| 20170803 | 14 | 240 | 24 | 45.28 | 71.43 | 88.53 |
| 20170803 | 15 | 250 | 24 | 47.44 | 74.40 | 89.04 |
| 20170803 | 16 | 80 | 24 | 14.87 | 23.81 | 87.23 |
| 20170803 | 17 | 650 | 24 | 120.95 | 193.45 | 87.31 |
| 20170803 | 18 | 170 | 24 | 30.31 | 50.60 | 83.67 |
| 20170803 | 19 | 100 | 24 | 17.46 | 29.76 | 81.94 |
| 20170803 | 20 | 330 | 24 | 82.05 | 98.21 | 116.67 |
| 20170803 | 21 | 220 | 24 | 35.68 | 65.48 | 76.10 |
| 20170803 | 22 | 210 | 24 | 34.91 | 62.50 | 78.00 |
| 20170803 | 23 | 200 | 24 | 34.28 | 59.52 | 80.42 |
| 20170803 | 24 | 120 | 24 | 18.41 | 35.71 | 71.98 |
| 20170803 | 25 | 60 | 24 | 8.68 | 17.86 | 67.85 |
| 20170803 | 26 | 0 | 24 | 0 | 0 | 0 |
| 20170816 | 1 | 140 | 24 | 18.71 | 41.67 | 62.72 |
| 20170816 | 2 | 120 | 24 | 17.53 | 35.71 | 68.55 |
| 20170816 | 3 | 200 | 24 | 32.52 | 59.52 | 76.31 |
| 20170816 | 4 | 90 | 24 | 13.81 | 26.79 | 72.00 |
| 20170816 | 5 | 70 | 24 | 10.15 | 20.83 | 68.03 |
| 20170816 | 6 | 100 | 24 | 16.79 | 29.76 | 78.77 |
| 20170816 | 7 | 100 | 24 | 17.82 | 29.76 | 83.60 |
| 20170816 | 8 | 120 | 24 | 21.76 | 35.71 | 85.09 |
| 20170816 | 9 | 190 | 24 | 37.16 | 56.55 | 91.76 |
| 20170816 | 10 | 260 | 24 | 50.82 | 77.38 | 91.71 |
| 20170816 | 11 | 170 | 24 | 33.95 | 50.60 | 93.70 |
| 20170816 | 12 | 370 | 24 | 76.42 | 110.12 | 96.92 |
| 20170816 | 13 | 450 | 24 | 91.62 | 133.93 | 95.54 |
| 20170816 | 14 | 190 | 24 | 39.31 | 56.55 | 97.09 |
| 20170816 | 15 | 200 | 24 | 40.87 | 59.52 | 95.88 |
| 20170816 | 16 | 230 | 24 | 46.56 | 68.45 | 94.99 |
| 20170816 | 17 | 280 | 24 | 136.82 | 193.45 | 98.77 |
| 20170816 | 18 | 70 | 24 | 13.76 | 20.83 | 92.26 |
| 20170816 | 19 | 130 | 24 | 25.00 | 38.69 | 90.24 |
| 20170816 | 20 | 230 | 24 | 45.62 | 68.45 | 93.08 |
| 20170816 | 21 | 150 | 24 | 29.38 | 44.64 | 91.89 |
| 20170816 | 22 | 140 | 24 | 26.38 | 41.67 | 88.42 |
| 20170816 | 23 | 220 | 24 | 43.19 | 65.48 | 92.11 |
| 20170816 | 24 | 200 | 24 | 39.22 | 59.52 | 92.01 |
| 20170816 | 25 | 60 | 24 | 10.03 | 17.86 | 78.40 |
| 20170816 | 26 | 0 | 24 | 0 | 0 | 0 |
| 20170901 | 1 | 250 | 24 | 34.64 | 74.40 | 65.01 |
| 20170901 | 2 | 130 | 24 | 19.44 | 38.69 | 70.19 |
| 20170901 | 3 | 360 | 24 | 56.63 | 107.14 | 73.81 |
| 20170901 | 4 | 110 | 24 | 17.52 | 32.74 | 74.74 |
| 20170901 | 5 | 160 | 24 | 26.74 | 47.62 | 78.41 |
| 20170901 | 6 | 200 | 24 | 34.25 | 59.52 | 80.35 |
| 20170901 | 7 | 140 | 24 | 25.81 | 41.67 | 86.51 |
| 20170901 | 8 | 130 | 24 | 22.22 | 38.69 | 80.20 |
| 20170901 | 9 | 170 | 24 | 32.83 | 50.60 | 90.61 |
| 20170901 | 10 | 310 | 24 | 59.05 | 92.26 | 89.38 |
| 20170901 | 11 | 200 | 24 | 39.58 | 59.52 | 92.85 |
| 20170901 | 12 | 110 | 24 | 21.66 | 32.74 | 92.38 |
| 20170901 | 13 | 290 | 24 | 58.94 | 86.31 | 95.37 |
| 20170901 | 14 | 130 | 24 | 25.53 | 38.69 | 92.15 |
| 20170901 | 15 | 290 | 24 | 59.16 | 86.31 | 95.73 |
| 20170901 | 16 | 290 | 24 | 56.87 | 86.31 | 92.01 |
| 20170901 | 17 | 290 | 24 | 131.51 | 86.31 | 94.94 |
| 20170901 | 18 | 220 | 24 | 43.75 | 65.48 | 93.32 |
| 20170901 | 19 | 270 | 24 | 53.82 | 80.36 | 93.54 |
| 20170901 | 20 | 280 | 24 | 54.83 | 83.33 | 91.88 |
| 20170901 | 21 | 350 | 24 | 70.04 | 104.17 | 93.91 |
| 20170901 | 22 | 200 | 24 | 37.36 | 59.52 | 87.66 |
| 20170901 | 23 | 200 | 24 | 37.88 | 59.52 | 88.87 |
| 20170901 | 24 | 280 | 24 | 53.22 | 83.33 | 89.19 |
| 20170901 | 25 | 160 | 24 | 28.41 | 47.62 | 83.32 |
| 20170901 | 26 | 60 | 24 | 8.72 | 17.86 | 68.18 |
| 20170917 | 1 | 0 | 24 | 0 | 0 | 0 |
| 20170917 | 2 | 380 | 24 | 60.11 | 113.10 | 74.23 |
| 20170917 | 3 | 130 | 24 | 23.92 | 38.69 | 86.34 |
| 20170917 | 4 | 120 | 24 | 22.09 | 35.71 | 86.36 |
| 20170917 | 5 | 140 | 24 | 26.67 | 41.67 | 89.40 |
| 20170917 | 6 | 140 | 24 | 25.78 | 41.67 | 86.40 |
| 20170917 | 7 | 150 | 24 | 26.80 | 44.64 | 83.82 |
| 20170917 | 8 | 210 | 24 | 38.62 | 62.50 | 86.30 |
| 20170917 | 9 | 160 | 24 | 25.26 | 47.62 | 74.07 |
| 20170917 | 10 | 310 | 24 | 58.43 | 92.26 | 88.44 |
| 20170917 | 11 | 300 | 24 | 56.58 | 89.29 | 88.50 |
| 20170917 | 12 | 210 | 24 | 38.68 | 62.50 | 86.43 |
| 20170917 | 13 | 80 | 24 | 14.49 | 23.81 | 84.98 |
| 20170917 | 14 | 150 | 24 | 28.51 | 44.64 | 89.19 |
| 20170917 | 15 | 270 | 24 | 53.06 | 80.36 | 92.22 |
| 20170917 | 16 | 150 | 24 | 26.45 | 44.64 | 82.73 |
| 20170917 | 17 | 250 | 24 | 130.23 | 74.40 | 94.01 |
| 20170917 | 18 | 180 | 24 | 33.91 | 53.57 | 88.39 |
| 20170917 | 19 | 140 | 24 | 25.43 | 41.67 | 85.22 |
| 20170917 | 20 | 140 | 24 | 26.07 | 41.67 | 87.38 |
| 20170917 | 21 | 220 | 24 | 36.14 | 65.48 | 77.07 |
| 20170917 | 22 | 130 | 24 | 21.21 | 38.69 | 76.57 |
| 20170917 | 23 | 90 | 24 | 12.78 | 26.79 | 66.63 |
| 20170917 | 24 | 60 | 24 | 8.87 | 17.86 | 69.39 |
| 20170917 | 25 | 0 | 24 | 0 | 0 | 0 |
| 20171001 | 1 | 230 | 24 | 34.25 | 68.45 | 69.87 |
| 20171001 | 2 | 480 | 24 | 91.44 | 142.86 | 89.38 |
| 20171001 | 3 | 260 | 24 | 47.95 | 77.38 | 86.54 |
| 20171001 | 4 | 170 | 24 | 31.94 | 50.60 | 88.17 |
| 20171001 | 5 | 350 | 24 | 62.39 | 104.17 | 83.65 |
| 20171001 | 6 | 160 | 24 | 30.61 | 47.17 | 89.76 |
| 20171001 | 7 | 210 | 24 | 40.96 | 62.50 | 91.53 |
| 20171001 | 8 | 380 | 24 | 74.33 | 113.10 | 91.78 |
| 20171001 | 9 | 240 | 24 | 45.66 | 71.43 | 89.28 |
| 20171001 | 10 | 250 | 24 | 48.82 | 74.40 | 91.63 |
| 20171001 | 11 | 200 | 24 | 32.20 | 59.52 | 75.54 |
| 20171001 | 12 | 530 | 24 | 96.89 | 157.74 | 85.78 |
| 20171001 | 13 | 170 | 24 | 31.49 | 50.60 | 86.92 |
| 20171001 | 14 | 290 | 24 | 57.26 | 86.31 | 92.66 |
| 20171001 | 15 | 180 | 24 | 34.88 | 53.57 | 90.93 |
| 20171001 | 16 | 190 | 24 | 121.82 | 56.55 | 87.94 |
| 20171001 | 17 | 260 | 24 | 51.77 | 77.38 | 93.44 |
| 20171001 | 18 | 120 | 24 | 22.32 | 35.71 | 87.27 |
| 20171001 | 19 | 190 | 24 | 34.01 | 56.55 | 83.99 |
| 20171001 | 20 | 250 | 24 | 46.61 | 74.40 | 87.48 |
| 20171001 | 21 | 150 | 24 | 26.69 | 44.64 | 83.48 |
| 20171001 | 22 | 500 | 24 | 91.63 | 148.81 | 85.99 |
| 20171001 | 23 | 140 | 24 | 23.13 | 41.67 | 77.53 |
| 20171001 | 24 | 100 | 24 | 12.75 | 29.76 | 59.81 |
| 20171017 | 1 | 100 | 24 | 12.24 | 29.76 | 57.41 |
| 20171017 | 2 | 210 | 24 | 36.61 | 62.50 | 81.79 |
| 20171017 | 3 | 250 | 24 | 45.14 | 74.40 | 84.72 |
| 20171017 | 4 | 130 | 24 | 23.76 | 38.69 | 85.77 |
| 20171017 | 5 | 100 | 24 | 18.21 | 29.76 | 85.43 |
| 20171017 | 6 | 90 | 24 | 16.07 | 26.79 | 83.76 |
| 20171017 | 7 | 90 | 24 | 15.54 | 26.79 | 81.03 |
| 20171017 | 8 | 140 | 24 | 24.56 | 41.67 | 82.31 |
| 20171017 | 9 | 200 | 24 | 33.56 | 59.52 | 78.74 |
| 20171017 | 10 | 80 | 24 | 16.74 | 23.81 | 98.19 |
| 20171017 | 11 | 130 | 24 | 24.84 | 38.69 | 89.66 |
| 20171017 | 12 | 130 | 24 | 25.80 | 38.69 | 93.11 |
| 20171017 | 13 | 70 | 24 | 13.54 | 20.83 | 90.74 |
| 20171017 | 14 | 100 | 24 | 18.81 | 29.76 | 88.24 |
| 20171017 | 15 | 420 | 24 | 130.83 | 125.00 | 94.44 |
| 20171017 | 16 | 100 | 24 | 19.32 | 29.76 | 90.67 |
| 20171017 | 17 | 140 | 24 | 27.22 | 41.67 | 91.23 |
| 20171017 | 18 | 150 | 24 | 27.70 | 44.64 | 86.66 |
| 20171017 | 19 | 190 | 24 | 34.94 | 56.55 | 86.28 |
| 20171017 | 20 | 200 | 24 | 36.79 | 59.52 | 86.32 |
| 20171017 | 21 | 90 | 24 | 16.95 | 26.79 | 88.36 |
| 20171017 | 22 | 130 | 24 | 23.78 | 38.69 | 85.84 |
| 20171017 | 23 | 160 | 24 | 29.63 | 47.62 | 86.89 |
| 20171105 | 1 | 0 | 24 | 0 | 0 | 0 |
| 20171105 | 2 | 280 | 24 | 43.43 | 83.33 | 72.78 |
| 20171105 | 3 | 290 | 24 | 54.39 | 86.31 | 88.01 |
| 20171105 | 4 | 260 | 24 | 49.56 | 77.38 | 89.44 |
| 20171105 | 5 | 180 | 24 | 35.64 | 53.57 | 92.90 |
| 20171105 | 6 | 130 | 24 | 24.41 | 38.69 | 88.11 |
| 20171105 | 7 | 140 | 24 | 26.91 | 41.67 | 90.19 |
| 20171105 | 8 | 160 | 24 | 30.60 | 47.62 | 89.73 |
| 20171105 | 9 | 350 | 24 | 69.19 | 104.17 | 92.76 |
| 20171105 | 10 | 510 | 24 | 90.99 | 151.79 | 83.71 |
| 20171105 | 11 | 110 | 24 | 20.08 | 32.74 | 85.67 |
| 20171105 | 12 | 250 | 24 | 50.29 | 74.40 | 94.39 |
| 20171105 | 13 | 130 | 24 | 30.36 | 38.69 | 109.58 |
| 20171105 | 14 | 520 | 24 | 104.39 | 154.76 | 94.47 |
| 20171105 | 15 | 460 | 24 | 90.67 | 136.90 | 92.49 |
| 20171105 | 16 | 260 | 24 | 129.55 | 77.38 | 93.52 |
| 20171105 | 17 | 140 | 24 | 27.62 | 41.67 | 92.57 |
| 20171105 | 18 | 280 | 24 | 53.70 | 83.33 | 89.99 |
| 20171105 | 19 | 480 | 24 | 93.78 | 142.86 | 91.67 |
| 20171105 | 20 | 200 | 24 | 39.31 | 59.52 | 92.22 |
| 20171105 | 21 | 510 | 24 | 96.97 | 151.79 | 89.22 |
| 20171105 | 22 | 110 | 24 | 20.34 | 32.74 | 86.75 |
| 20171105 | 23 | 220 | 24 | 39.40 | 65.48 | 84.04 |
| 20171105 | 24 | 190 | 24 | 32.63 | 56.55 | 80.59 |

**Note:** The sampling locations were numbered from the north bank to the south bank of the NW transect.

**Supplementary Table S6.** The measured CH_4_ emission fluxes by the floating chambers at the 5 sampling points of NE transect in 2015

| Dates | Sampling points | Chamber 1 | Chamber 2 | Chamber 3 |
| --- | --- | --- | --- | --- |
| 20150108 | NEP1 | 0.082 | 0.073 | 0.055 |
| 20150108 | NEP2 | 0.065 | 0.077 | 0.046 |
| 20150108 | NEP3 | 0.058 | 0.074 | 0.068 |
| 20150108 | NEP4 | 0.089 | 0.075 | 0.053 |
| 20150108 | NEP5 | 0.027 | 0.022 | 0.032 |
| 20150123 | NEP1 | No data | 0.058 | No data |
| 20150123 | NEP2 | 0.049 | 0.031 | 0.063 |
| 20150123 | NEP3 | 0.030 | No data | 0.022 |
| 20150123 | NEP4 | No data | 0.053 | No data |
| 20150123 | NEP5 | 0.099 | 0.077 | 0.083 |
| 20150205 | NEP1 | 0.086 | 0.072 | No data |
| 20150205 | NEP3 | 0.063 | 0.082 | 0.025 |
| 20150205 | NEP4 | 0.048 | 0.047 | No data |
| 20150205 | NEP5 | 0.046 | 0.030 | 0.025 |
| 20150313 | NEP1 | 0.0071 | 0.0057 | No data |
| 20150313 | NEP2 | 0.041 | 0.011 | 0.013 |
| 20150313 | NEP4 | No data | 0.015 | No data |
| 20150313 | NEP5 | 0.012 | 0.014 | 0.009 |
| 20150416 | NEP1 | 0.0011 | 0.0079 | 0.0041 |
| 20150416 | NEP2 | 0.023 | 0.027 | 0.055 |
| 20150416 | NEP3 | 0.033 | 0.068 | 0.037 |
| 20150416 | NEP4 | 0.084 | 0.096 | 0.106 |
| 20150416 | NEP5 | 0.136 | 0.125 | 0.106 |
| 20150521 | NEP1 | 0.038 | 0.027 | 0.039 |
| 20150521 | NEP2 | 0.066 | 0.057 | 0.039 |
| 20150521 | NEP3 | 0.069 | 0.024 | 0.062 |
| 20150521 | NEP4 | 0.094 | 0.040 | 0.075 |
| 20150521 | NEP5 | 0.149 | 0.057 | 0.128 |
| 20150629 | NEP1 | No data | 0.057 | 0.039 |
| 20150629 | NEP2 | 0.140 | 0.083 | No data |
| 20150629 | NEP3 | 0.040 | 0.105 | 0.057 |
| 20150629 | NEP4 | 0.016 | 0.027 | 0.024 |
| 20150629 | NEP5 | 0.016 | 0.048 | 0.048 |
| 20150727 | NEP1 | 0.044 | 0.07 | 0.044 |
| 20150727 | NEP2 | 0.049 | 0.052 | 0.034 |
| 20150727 | NEP3 | 0.082 | 0.051 | 0.065 |
| 20150727 | NEP4 | 0.048 | 0.059 | 0.040 |
| 20150727 | NEP5 | 0.032 | 0.040 | 0.060 |
| 20150820 | NEP1 | 0.121 | 0.143 | 0.103 |
| 20150820 | NEP2 | 0.101 | 0.097 | 0.108 |
| 20150820 | NEP3 | 0.120 | 0..99 | 0.100 |
| 20150820 | NEP4 | 0.093 | 0.110 | 0.081 |
| 20150820 | NEP5 | 0.100 | 0.110 | 0.070 |
| 20150922 | NEP1 | 0.100 | 0.120 | 0.096 |
| 20150922 | NEP2 | 0.120 | 0.100 | 0.160 |
| 20150922 | NEP3 | 0.084 | 0.109 | 0.110 |
| 20150922 | NEP4 | 0.087 | 0.140 | 0.091 |
| 20150922 | NEP5 | 0.062 | 0.090 | 0.095 |
| 20151026 | NEP1 | 0.342 | No data | 0.096 |
| 20151026 | NEP2 | 0.055 | 0.043 | 0.075 |
| 20151026 | NEP3 | 0.039 | 0.052 | 0.074 |
| 20151026 | NEP4 | 0.090 | 0.144 | 0.095 |
| 20151026 | NEP5 | 0.067 | 0.130 | 0.097 |
| 20151204 | NEP1 | 0.390 | 0.243 | No data |
| 20151204 | NEP2 | 0.085 | 0.120 | 0.110 |
| 20151204 | NEP3 | 0.120 | 0.116 | 0.065 |
| 20151204 | NEP4 | 0.100 | 0.120 | 0.080 |
| 20151204 | NEP5 | 0.060 | 0.080 | 0.087 |
| 20151226 | NEP1 |  | 0.030 | 0.094 |
| 20151226 | NEP2 | 0.069 | 0.052 | 0.077 |
| 20151226 | NEP3 | 0.060 | 0.065 | 0.072 |
| 20151226 | NEP4 | 0.046 | 0.084 | 0.070 |
| 20151226 | NEP5 | 0.047 | 0.054 | 0.070 |

Note: The unit of CH_4_ flux is mg CH_4_ m^-2^ h^-1^. The sampling points from NEP1 to NEP5 distributed from the margin to pelagic zones.

**Supplementary Table S7.** Complete dataset of the measured CH_4_ emission fluxes by the floating chambers at the 5 sampling points of SW transect from Dec. 2014 to Dec. 2015

| Dates | Sampling points | Chamber 1 | Chamber 2 | Chamber 3 |
| --- | --- | --- | --- | --- |
| 20141206 | SWP1 | 0.290 | 0.200 | 0.260 |
| 20141206 | SWP2 | 0.200 | 0.170 | 0.190 |
| 20141206 | SWP3 | 0.220 | 0.170 | 0.260 |
| 20141206 | SWP4 | 0.240 | 0.300 | 0.250 |
| 20141206 | SWP5 | 0.140 | 0.100 | 0.110 |
| 20150104 | SWP1 | 0.049 | 0.029 | 0.048 |
| 20150104 | SWP2 | 0.120 | 0.100 | 0.100 |
| 20150104 | SWP4 | 0.063 | 0.049 | 0.052 |
| 20150104 | SWP5 | 0.016 | 0.026 | 0.016 |
| 20150120 | SWP1 | 0.038 | 0.140 | No data |
| 20150120 | SWP2 | No data | 0.140 | 0.083 |
| 20150120 | SWP3 | 0.100 | 0.03 | 0.077 |
| 20150120 | SWP4 | 0.210 | 0.170 | 0.360 |
| 20150120 | SWP5 | 0.120 | 0.130 | No data |
| 20150208 | SWP2 | 0.280 | 0.240 | 0.180 |
| 20150208 | SWP4 | 0.220 | 0.230 | 0.140 |
| 20150208 | SWP5 | 0.043 | 0.053 | 0.050 |
| 20150326 | SWP1 | 0.110 | 0.082 | 0.071 |
| 20150326 | SWP3 | 0.110 | 0.100 | No data |
| 20150326 | SWP4 | 0.048 | 0.089 | 0.100 |
| 20150326 | SWP5 | No data | 0.066 | 0.110 |
| 20150417 | SWP1 | 0.056 | 0.043 | 0.048 |
| 20150417 | SWP2 | 0.100 | No data | 0.036 |
| 20150417 | SWP3 | 0.046 | 0.083 | 0.057 |
| 20150417 | SWP5 | No data | 0.070 | 0.085 |
| 20150602 | SWP1 | 0.130 | 0.034 | 0.047 |
| 20150602 | SWP2 | 0.025 | 0.015 | 0.032 |
| 20150602 | SWP3 | 0.036 | 0.028 | 0.041 |
| 20150602 | SWP4 | 0.023 | 0.031 | 0.029 |
| 20150602 | SWP5 | 0.030 | No data | 0.020 |
| 20150625 | SWP1 | 0.031 | 0.039 | 0.028 |
| 20150625 | SWP2 | 0.043 | 0.019 | 0.040 |
| 20150625 | SWP3 | 0.027 | 0.016 | 0.021 |
| 20150625 | SWP4 | 0.015 | 0.014 | 0.017 |
| 20150625 | SWP5 | 0.031 | 0.012 | 0.014 |
| 20150801 | SWP1 | 0.520 | 0.550 | 0.350 |
| 20150801 | SWP2 | 0.300 | 0.290 | 0.400 |
| 20150801 | SWP3 | 0.110 | 0.087 | 0.099 |
| 20150801 | SWP4 | 0.100 | 0.130 | 0.083 |
| 20150801 | SWP5 | 0.082 | 0.078 | 0.072 |
| 20150901 | SWP1 | 0.100 | 0.089 | 0.072 |
| 20150901 | SWP2 | 0.140 | 0.110 | 0.083 |
| 20150901 | SWP3 | 0.083 | 0.050 | 0.022 |
| 20150901 | SWP4 | 0.088 | 0.110 | 0.066 |
| 20150901 | SWP5 | 0.062 | 0.040 | 0.091 |
| 20151003 | SWP1 | 0.130 | 0.300 | 0.230 |
| 20151003 | SWP2 | 0.170 | 0.150 | 0.120 |
| 20151003 | SWP3 | 0.230 | 0.040 | 0.050 |
| 20151003 | SWP4 | 0.030 | 0.041 | 0.045 |
| 20151003 | SWP5 | 0.078 | 0.045 | 0.063 |
| 20151105 | SWP1 | 0.055 | 0.040 | 0.069 |
| 20151105 | SWP2 | 0.076 | 0.150 | 0.078 |
| 20151105 | SWP3 | 0.144 | 0.160 | 0.140 |
| 20151105 | SWP4 | 0.110 | 0.120 | 0.069 |
| 20151105 | SWP5 | 0.120 | 0.160 | 0.130 |
| 20151128 | SWP1 | 0.071 | 0.099 | No data |
| 20151128 | SWP2 | 0.240 | 0.270 | 0.200 |
| 20151128 | SWP3 | 0.11 | 0.099 | 0.085 |
| 20151128 | SWP4 | 0.086 | 0.100 | 0.086 |
| 20151128 | SWP5 | 0.073 | 0.230 | 0.058 |
| 20151227 | SWP1 | 0.100 | 0.140 | 0.079 |
| 20151227 | SWP2 | 0.100 | 0.095 | 0.054 |
| 20151227 | SWP3 | 0.050 | 0.073 | 0.040 |
| 20151227 | SWP4 | 0.067 | 0.078 | 0.039 |
| 20151227 | SWP5 | 0.039 | 0.054 | 0.027 |

Note: The unit of CH_4_ emission flux is mg CH_4_ m^-2^ h^-1^. The sampling points from SWP1 to SWP5 distributed from the margin to pelagic zones.

**Supplementary Table S8.** Complete dataset of the measured CH_4_ emission fluxes by the floating chambers at the 5 sampling points of SE transect from Jan. 2015 to Jan. 2016.

| Dates | Sampling points | Chamber 1 | Chamber 2 | Chamber 3 |
| --- | --- | --- | --- | --- |
| 20141123 | SEP1 | 0.048 | 0.028 | 0.030 |
| 20141123 | SEP2 | 0.038 | 0.120 | 0.160 |
| 20141123 | SEP3 | 0.054 | 0.053 | 0.059 |
| 20141123 | SEP4 | 0.049 | 0.061 | 0.045 |
| 20150105 | SEP1 | 0.046 | 0.026 | 0.053 |
| 20150105 | SEP2 | 0.063 | 0.044 | 0.047 |
| 20150105 | SEP3 | 0.089 | 0.049 | 0.056 |
| 20150105 | SEP4 | 0.098 | 0.064 | 0.068 |
| 20150121 | SEP1 | 0.053 | 0.053 | 0.049 |
| 20150121 | SEP2 | 0.046 | No data | No data |
| 20150121 | SEP3 | 0.030 | 0.040 | 0.043 |
| 20150121 | SEP4 | 0.150 | 0.100 | 0.130 |
| 20150121 | SEP5 | 0.170 | No data | No data |
| 20150207 | SEP1 | 0.057 | 0.043 | 0.054 |
| 20150207 | SEP2 | 0.059 | 0.043 | 0.043 |
| 20150207 | SEP3 | 0.010 | 0.024 | 0.013 |
| 20150207 | SEP4 | 0.023 | 0.020 | 0.019 |
| 20150207 | SEP5 | 0.034 | 0.029 | 0.030 |
| 20150319 | SEP1 | 0.015 | 0.027 | 0.015 |
| 20150319 | SEP2 | 0.063 | 0.073 | 0.052 |
| 20150319 | SEP3 | 0.037 | 0.028 | 0.048 |
| 20150319 | SEP4 | No data | 0.019 | 0.015 |
| 20150415 | SEP1 | 0.036 | 0.068 | 0.031 |
| 20150415 | SEP2 | 0.028 | No data | 0.007 |
| 20150415 | SEP3 | 0.035 | No data | 0.030 |
| 20150415 | SEP4 | 0.027 | 0.050 | 0.038 |
| 20150415 | SEP5 | 0.044 | 0.021 | 0.029 |
| 20150601 | SEP1 | 0.074 | 0.075 | 0.086 |
| 20150601 | SEP2 | 0.046 | 0.033 | 0.044 |
| 20150601 | SEP3 | 0.096 | 0.061 | 0.063 |
| 20150601 | SEP4 | 0.047 | 0.035 | 0.045 |
| 20150601 | SEP5 | 0.030 | 0.030 | 0.055 |
| 20150630 | SEP1 | 0.048 | 0.029 | 0.053 |
| 20150630 | SEP2 | 0.049 | 0.060 | 0.054 |
| 20150630 | SEP3 | 0.077 | 0.056 | 0.053 |
| 20150630 | SEP4 | 0.083 | 0.046 | 0.074 |
| 20150630 | SEP5 | 0.110 | 0.099 | 0.170 |
| 20150726 | SEP1 | 0.067 | 0.016 | 0.036 |
| 20150726 | SEP2 | 0.024 | 0.042 | 0.019 |
| 20150726 | SEP3 | 0.033 | 0.033 | 0.047 |
| 20150726 | SEP4 | 0.081 | 0.090 | 0.048 |
| 20150726 | SEP5 | 0.091 | 0.064 | 0.048 |
| 20150902 | SEP1 | 0.088 | 0.049 | 0.039 |
| 20150902 | SEP2 | 0.120 | 0.043 | 0.074 |
| 20150902 | SEP3 | 0.070 | 0.077 | 0.098 |
| 20150902 | SEP4 | 0.039 | 0.036 | 0.031 |
| 20150902 | SEP5 | 0.088 | 0.059 | 0.099 |
| 20151009 | SEP1 | 0.088 | 0.031 | 0.083 |
| 20151009 | SEP2 | 0.054 | 0.059 | 0.074 |
| 20151009 | SEP3 | 0.072 | 0.076 | 0.081 |
| 20151009 | SEP4 | 0.071 | 0.075 | 0.100 |
| 20151009 | SEP5 | 0.056 | 0.081 | 0.048 |
| 20151106 | SEP1 | 0.310 | 0.120 | 0.100 |
| 20151106 | SEP2 | 0.098 | 0.120 | 0.067 |
| 20151106 | SEP3 | 0.053 | 0.076 | 0.072 |
| 20151106 | SEP4 | 0.056 | 0.096 | No data |
| 20151106 | SEP5 | 0.058 | 0.058 | 0.053 |
| 20151203 | SEP1 | 0.053 | 0.084 | 0.120 |
| 20151203 | SEP2 | 0.060 | 0.063 | 0.250 |
| 20151203 | SEP3 | 0.085 | 0.100 | No data |
| 20151203 | SEP4 | 0.059 | 0.078 | 0.078 |
| 20151203 | SEP5 | 0.058 | 0.057 | 0.071 |
| 20160108 | SEP1 | 0.057 | 0.081 | 0.087 |
| 20160108 | SEP2 | 0.110 | 0.052 | 0.064 |
| 20160108 | SEP3 | 0.170 | 0.120 | 0.130 |
| 20160108 | SEP4 | 0.075 | 0.110 | 0.085 |
| 20160108 | SEP5 | 0.130 | 0.095 | 0.090 |

Note: The unit of CH_4_ emission flux is mg CH_4_ m^-2^ h^-1^. The sampling points from SEP1 to SEP5 distributed from the margin to pelagic zones.

**Supplementary Table S9.** Complete dataset of the measured CH_4_ emission fluxes by the floating chambers at the 4 sampling points of downstream river from Dec. 2014 to Dec. 2015

| Dates | Sampling points | Chamber 1 | Chamber 2 | Chamber 3 |
| --- | --- | --- | --- | --- |
| 20141207 | DRP1 | 2.67 | 1.56 | 1.04 |
| 20141207 | DRP2 | 1.68 | 1.09 | 0.93 |
| 20141207 | DRP3 | 2.00 | 1.29 | 0.82 |
| 20141207 | DRP4 | 1.97 | 1.37 | 0.96 |
| 20150107 | DRP1 | 1.72 | 1.04 | 1.42 |
| 20150107 | DRP2 | 0.53 | 0.43 | 0.58 |
| 20150107 | DRP3 | 0.91 | 1.12 | 0.98 |
| 20150107 | DRP4 | 1.47 | 0.35 | 0.97 |
| 20150122 | DRP1 | 1.31 | 0.99 | 1.33 |
| 20150122 | DRP2 | 1.04 | 0.50 | 0.82 |
| 20150122 | DRP3 | 1.46 | 0.54 | No data |
| 20150122 | DRP4 | 1.50 | 1.22 | 0.75 |
| 20150206 | DRP1 | 0.46 | 0.24 | 0.25 |
| 20150206 | DRP2 | 0.20 | 0.19 | 0.17 |
| 20150206 | DRP3 | 0.11 | 0.07 | 0.16 |
| 20150206 | DRP4 | 0.064 | 0.095 | 0.078 |
| 20150325 | DRP1 | 0.27 | 0.38 | 0.36 |
| 20150325 | DRP2 | 0.43 | 0.42 | 0.30 |
| 20150325 | DRP3 | 0.31 | 0.28 | 0.35 |
| 20150325 | DRP4 | 0.076 | 0.077 | 0.10 |
| 20150414 | DRP1 | 0.90 | 0.86 | 1.13 |
| 20150414 | DRP2 | 0.23 | 0.19 | 0.22 |
| 20150414 | DRP3 | 0.40 | 0.68 | 0.45 |
| 20150414 | DRP4 | 0.073 | 0.12 | 0.091 |
| 20150522 | DRP1 | 0.56 | 1.43 | 0.48 |
| 20150522 | DRP2 | 0.13 | 0.11 | 0.11 |
| 20150522 | DRP3 | 0.31 | 0.59 | 0.31 |
| 20150522 | DRP4 | 0.043 | 0.037 | 0.056 |
| 20150703 | DRP1 | 0.63 | 0.29 | 0.36 |
| 20150703 | DRP2 | 0.088 | 0.079 | 0.16 |
| 20150703 | DRP3 | 0.085 | 0.080 | 0.084 |
| 20150703 | DRP4 | 0.26 | 0.27 | 0.25 |
| 20150731 | DRP1 | 0.33 | 0.49 | No data |
| 20150731 | DRP2 | 0.17 | 0.10 | 0.11 |
| 20150731 | DRP3 | 0.20 | 0.07 | 0.21 |
| 20150731 | DRP4 | 0.13 | 0.16 | 0.058 |
| 20150825 | DRP1 | 0.77 | 0.29 | 0.39 |
| 20150825 | DRP2 | 0.074 | 0.13 | 0.081 |
| 20150825 | DRP3 | 0.14 | 0.063 | 0.093 |
| 20150825 | DRP4 | 0.22 | 0.19 | 0.20 |
| 20150918 | DRP1 | 0.27 | 0.67 | 0.68 |
| 20150918 | DRP2 | 0.12 | 0.11 | 0.071 |
| 20150918 | DRP3 | 0.16 | 0.14 | 0.19 |
| 20150918 | DRP4 | 0.13 | 0.15 | 0.33 |
| 20151016 | DRP1 | 0.77 | 0.58 | 1.03 |
| 20151016 | DRP2 | 0.15 | 0.12 | 0.14 |
| 20151016 | DRP3 | 0.12 | 0.11 | 0.11 |
| 20151016 | DRP4 | 0.31 | 0.27 | 0.32 |
| 20151126 | DRP1 | 0.53 | 0.91 | 1.39 |
| 20151126 | DRP2 | 0.18 | 0.12 | 0.14 |
| 20151126 | DRP3 | 0.21 | 0.42 | 0.90 |
| 20151126 | DRP4 | 0.50 | 0.23 | 0.74 |
| 20151228 | DRP1 | 0.32 | 0.72 | 0.38 |
| 20151228 | DRP2 | 1.03 | 0.085 | 0.078 |
| 20151228 | DRP3 | 0.085 | 0.15 | 0.13 |
| 20151228 | DRP4 | 0.12 | 0.19 | 1.57 |

Note: The unit of CH_4_ emission flux is mg CH_4_ m^-2^ h^-1^. The sampling points (DRP1-DRP4) have distance of 0.35, 1, 4, and 7 km away from the Xin’anjiang Dam.
